# Hepatitis Infection in the Treatment of Opioid Dependence and Abuse

**DOI:** 10.4137/sart.s580

**Published:** 2008-04-28

**Authors:** Thomas F Kresina, Diana Sylvestre, Leonard Seeff, Alain H Litwin, Kenneth Hoffman, Robert Lubran, H Westley Clark

**Affiliations:** 1 Center for Substance Abuse Treatment, Substance Abuse and Mental Health Services Administration, Rockville, MD.; 2 Department of Medicine, University of California, San Francisco and Organization to Achieve Solutions In Substance Abuse (O.A.S.I.S.) Oakland, CA.; 3 Division of Digestive Diseases and Nutrition, National Institute on Diabetes, Digestive, and Kidney Diseases, National Institutes of Health, DHHS, Bethesda, MD.; 4 Division of Substance Abuse, Albert Einstein College of Medicine, Montefiore Medical Center Bronx, NY.

**Keywords:** hepatitis, methadone, substance abuse treatment, medication assisted treatment

## Abstract

Many new and existing cases of viral hepatitis infections are related to injection drug use. Transmission of these infections can result directly from the use of injection equipment that is contaminated with blood containing the hepatitis B or C virus or through sexual contact with an infected individual. In the latter case, drug use can indirectly contribute to hepatitis transmission through the dis-inhibited at-risk behavior, that is, unprotected sex with an infected partner. Individuals who inject drugs are at-risk for infection from different hepatitis viruses, hepatitis A, B, or C. Those with chronic hepatitis B virus infection also face additional risk should they become co-infected with hepatitis D virus. Protection from the transmission of hepatitis viruses A and B is best achieved by vaccination. For those with a history of or who currently inject drugs, the medical management of viral hepatitis infection comprising screening, testing, counseling and providing care and treatment is evolving. Components of the medical management of hepatitis infection, for persons considering, initiating, or receiving pharmacologic therapy for opioid addiction include: testing for hepatitis B and C infections; education and counseling regarding at-risk behavior and hepatitis transmission, acute and chronic hepatitis infection, liver disease and its care and treatment; vaccination against hepatitis A and B infection; and integrative primary care as part of the comprehensive treatment approach for recovery from opioid abuse and dependence. In addition, participation in a peer support group as part of integrated medical care enhances treatment outcomes. Liver disease is highly prevalent in patient populations seeking recovery from opioid addiction or who are currently receiving pharmacotherapy for opioid addiction. Pharmacotherapy for opioid addiction is not a contraindication to evaluation, care, or treatment of liver disease due to hepatitis virus infection. Successful pharmacotherapy for opioid addiction stabilizes patients and improves patient compliance to care and treatment regimens as well as promotes good patient outcomes. Implementation and integration of effective hepatitis prevention programs, care programs, and treatment regimens in concert with the pharmacological therapy of opioid addiction can reduce the public health burdens of hepatitis and injection drug use.

## Viral Hepatitis Infections, Liver Disease and Injection Drug Use

Chronic liver disease is a major health problem with chronic liver disease and cirrhosis the thirteenth most frequent cause of death in the United States (CDC, 2007). Chronic liver disease can result from viral hepatitis infection. Hepatitis viruses can be transmitted from one individual to another through shared drug use practices, especially those involving injection of drugs. The disease burden and estimated incident infections of hepatitis A virus (HAV), hepatitis B virus (HBV) and hepatitis C virus (HCV) are summarized in Table 1 (CDC, 2006).

### Hepatitis A virus infection

HAV infection in injection drug users can have significant medical consequences. HAV infection in patients already chronically infected with HBV or HCV can result in fulminant liver failure. HAV also will increase the health risks faced by individuals with HIV infection, and a recent national study of human immunodeficiency virus (HIV)-infected individuals in primary care revealed that only 12.5 percent of injection drug users were vaccinated against HAV infection (Tedaldi et al. 2004).

**Figure 1:**
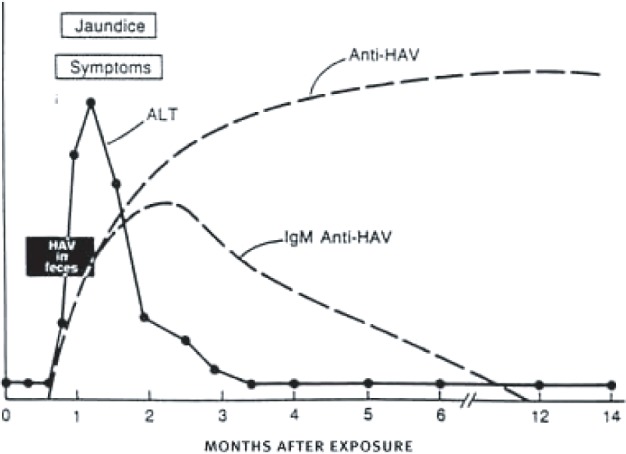
The time course of an uncomplicated HAV infection (from Larson et al. 2005).

Hepatitis A is a virus transmitted through the fecal-oral route, by consuming contaminated food or water that may have been fecally contaminated, or transmitted directly from person-to-person through poor hygiene and intimate contact. Hepatitis A has an incubation period ranging from 15 days to 50 days, and, in uncomplicated cases, the infection is completely resolved by 6 months after infection (Fig. 1). There is no specific treatment for HAV infection, and most people recover without medical intervention, although supportive measures such as intravenous fluids are occasionally needed.

As many as a third of all persons in the United States are estimated to have been infected at some time, usually during childhood. The estimated annual incidence of HAV infection has dropped substantially since introduction of an HAV vaccine in 1995, but computer models suggest an estimated annual incidence of 270,000 cases in the United States, more than 10 times the number actually reported (Armstrong and Bell, 2002).

Among injection drug users, HAV can be transmitted intravenously (though rarely) through shared equipment or HAV-contaminated water, but is far more commonly transmitted by the usual fecal-oral route through unhygienic practices during drug preparation and sharing. The fatality rate for HAV infection is generally low (less than 1 percent, or about 100 persons per year), although injection drug users with preexisting chronic liver disease (such as alcoholic liver disease or chronic HCV infection) are at increased risk of liver failure and death. Nearly 20 percent of reported HAV cases have occurred among injection drug users (CDC, 1999), and approximately 6 percent of reported HAV cases occurred among injection drug users during 2002 (CDC, 2004).

In a recent study of young injectors in Juneau, Alaska, 33 percent of those tested were seropositive for antibodies to HAV (Wells et al. 2006). Correlates of infection included having less than a high school education, exposure to HBV, and frequent opioid injection in the last 30 days. In a similar study from Canada, 58 percent of a large cohort of individuals entering opioid detoxification were HAV seropositive (Reimer et al. 2006). In another study of individuals entering a treatment program for opioid dependence, 41.2 percent were seropositive for HAV (Gerlich et al. 2006). Other epidemiologic studies in Europe have shown multiple outbreaks among men having sex with men of infection with a specific genotype of HAV (IA), indicating the exchange of HAV to endemic levels among groups with identifiable behaviors (Stene-Johansen et al. 2007).

HAV infection can be prevented by vaccination, and the Centers for Disease Control and Prevention (CDC) recommends that all injection drug users not previously vaccinated be immunized with the hepatitis A vaccine to protect from severe liver disease. Vaccination is not harmful for persons who have been infected with HAV, and thus pre-vaccinating testing to determine need for vaccine is not recommended unless people are in a stable environment (e.g. in-patient long term drug treatment) where it can be assured they will be around when the test result is received.

### Hepatitis B virus infection

**Figure 2:**
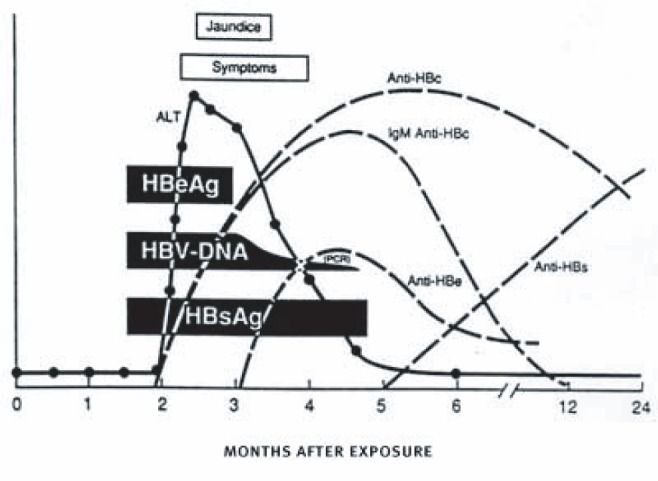
Time course of acute HBV infection showing the expression of viral markers of infection and the immune response to infection among persons who recover from acute infection (from Larson et al. 2005).

HBV infection typically is a self-limited illness, with infected adults recovering fully in approximately 6 months (Fig. 2). However, persons with chronic liver disease from other causes (e.g. chronic HCV infection) may be more likely to develop liver failure from acute HBV infection. Multiple hepatitis infections, or coinfections are common among injection drug users, particularly in the context of observed health disparities among African American and Hispanic drug injectors (Estrada, 2005; Fisher et al. 2006). Injection drug users who may already have underlying liver disease and become infected with HBV are at high risk for serious liver disease. In a series of case studies of injection drug users with acute HBV infection, nearly all those with underlying chronic HCV infection died from fulminant liver failure (Garfein et al. 2004). Thus, prevention of HBV infection among injection drug users is critically important, and the CDC recommends vaccination for all injection drug users who have not been previously vaccinated or known to have been exposed to HBV. Vaccination is not harmful for persons who have been infected with HBV, thus, pre-vaccination testing should not be a barrier to receiving vaccine.

Counseling and educating injection drug users about HBV infection and vaccination is also important, as studies have shown that a majority of injection drug users questioned were not able to accurately self-report their vaccination status (de la Fuente et al. 2007; Kuo et al. 2004a). Studies have shown that an HBV vaccination program targeting injection drug users is both feasible and effective (Altice et al. 2005; Burt et al. 2007; Kuo et al. 2004; Quaglio et al. 2002).

**Figure 3:**
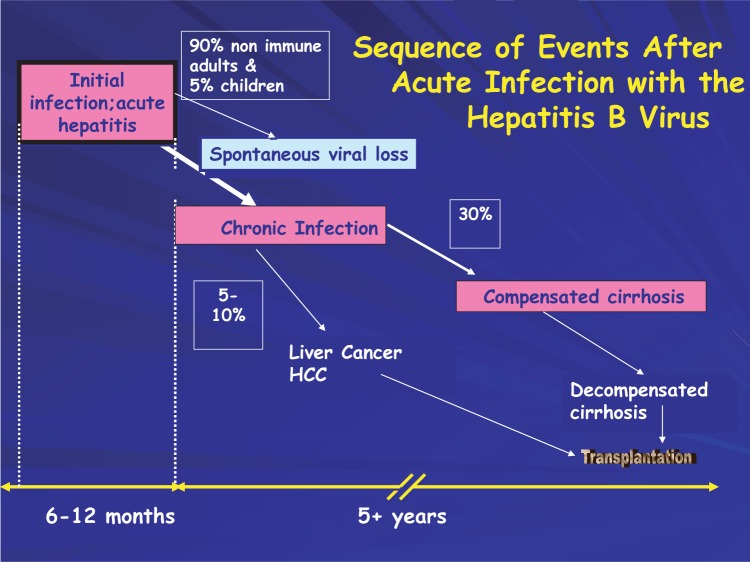
Diagram of the natural history of infection with HBV.

Roughly 5 percent of persons in the United States have been exposed to HBV, and an estimated 51,000 new cases of acute HBV infection occurred in 2005. Because of the success of infant and childhood HBV vaccination programs in the United States, the vast majority of acute HBV infections occur in adults. Most cases result in complete recovery and immunity from future infection. HBV infection may become chronic in only about 5 percent of persons infected as adults (Fig. 3). Chronic HBV infection affects the liver and may lead over time to cirrhosis (yearly incidence of 1.3 percent to 5.9 percent) that may result in liver failure or cancer. The 5-year survival rate of patients with HBV-related cirrhosis ranges from 52 percent to 82 percent. Co-infection with hepatitis D virus (HDV) or HIV or chronic alcohol consumption are the main factors that modify and exacerbate the course of liver disease in chronic infection (Sheng et al. 2007).

As seen in Table 1, more than one million persons in the United States are chronically infected with HBV. Roughly one in five persons with chronic HBV infection will die prematurely from the consequences of chronic liver disease. Approximately 4,000 persons die each year of HBV-related liver cirrhosis, and 1,500 individuals die of heptocellular carcinoma related to HBV infection each year (CDC, 2002; Fattovitich et al. 2004).

Although sexual contact with an individual chronically infected with HBV is the most common route of transmission, sharing injection drug use equipment contaminated with HBV can also lead to infection. Injection drug users accounted for approximately 12 percent of all cases in 2002, with 40 percent becoming infected with HBV after 1 year of injection drug use and more than 80 percent becoming infected after 10 years (CDC, 2002). Sexual transmission, accounting for half of all HBV infections (41 percent heterosexual, 9 percent men having sex with men; CDC, 2002), also may be a significant route of infection among addicted persons as a result of unsafe behavior, such as exchanging sex for drugs. Other risk factors associated with HBV infection include the presence of HCV co-infection and a history of imprisonment (Backmund et al. 2006).

#### Treatment of Hepatitis B virus infection

The current goals of treatment of chronic HBV infection are to achieve a sustained suppression of HBV replication and a remission of liver disease (Lok and McMahon, 2007). Reducing the progression of liver disease is important so that liver cancer, cirrhosis, and hepatic failure does not develop and reversing decompensated cirrhosis is important so that the patient is no longer a candidate for liver transplantation (AASLD, 2003; Fung and Lok, 2005; Kanwal et al. 2005). Factors that influence a response to treatment include patient age, severity of liver disease, likelihood of a treatment response, and comorbid complications. Interferon may be used as an initial therapy for a predefined time period, although six drugs can be used for the treatment of chronic HBV: interferon-alpha(2b), pegylated interferon-alpha(2a), lamivudine, adefovir, entecavir, and telbivudine (Ruiz-Sancho et al. 2007). Combination therapy, using two or more approved drugs for chronic HBV infection, is being investigated and may enhance the patient's response to treatment. Additional treatment complications may occur in Asian chronic HBV-infected patients who acquire the virus early in life (Yuen, 2007). Thus, it is important to introduce hepatitis education and prevention programs into a substance abuse treatment setting (Hagedorn et al. 2007; Strauss et al. 2007). For example, the Healthy Liver Program in Minnesota provides screening, education, and vaccination against hepatitis infection, particularly HBV.

### Hepatitis B virus and Hepatitis D virus co-infection

**Figure 4:**
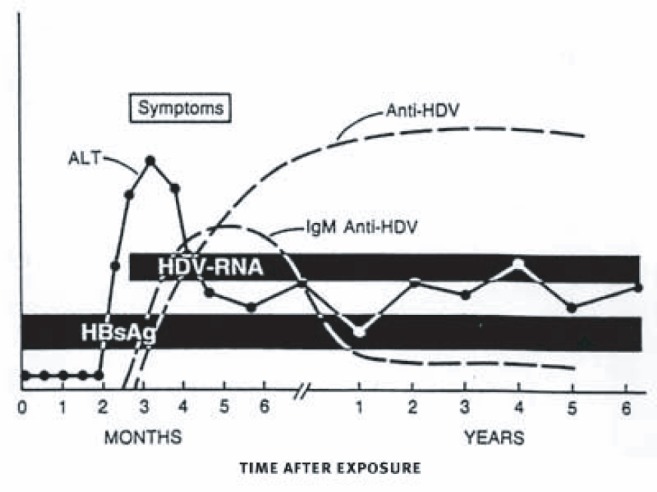
Time course of acute (months) and chronic (years) HDV superinfection (HBV/HDV co-infection) (from Larson et al. 2005).

New HBV infection may be accompanied by co-infection with HDV, which can replicate only with the aid of a “helper” function of HBV; persons who have chronic HBV infection can subsequently be infected with HDV (called “super-infection”) (Fig. 4). HDV co-infection should be suspected in any patient with severe acute HBV infection. The prevalence of HDV in the United States is relatively low, although injection drug users may be at high risk. HDV infection complicates the liver disease associated with HBV infection and increases the risk of liver cancer two- to sixfold compared to HBV infection alone (Fattovitch et al. 2004). In a recent outbreak of HBV infection in injection drug users with a high prevalence of HDV infection, risk factors for co-infection were having more than one sex partner, injecting more than four times a day, and sharing injection equipment with more than two persons. The important public health issue represented by HBV/HDV co-infection can be seen in this outbreak, as all co-infected individuals died of fulminant liver failure (Bialek et al. 2005).

Interferon therapy in persons co-infected with HBV and HDV is less effective because of the complications associated with this type of infection. While treatment decreases liver enzyme levels and suppresses HBV replication, the virus is not eradicated; typically, HBV infection reasserts itself if treatment is stopped. Initial prevention of HBV infection through vaccination will prevent subsequent infection with HDV.

### Hepatitis C virus infection

**Figure 5:**
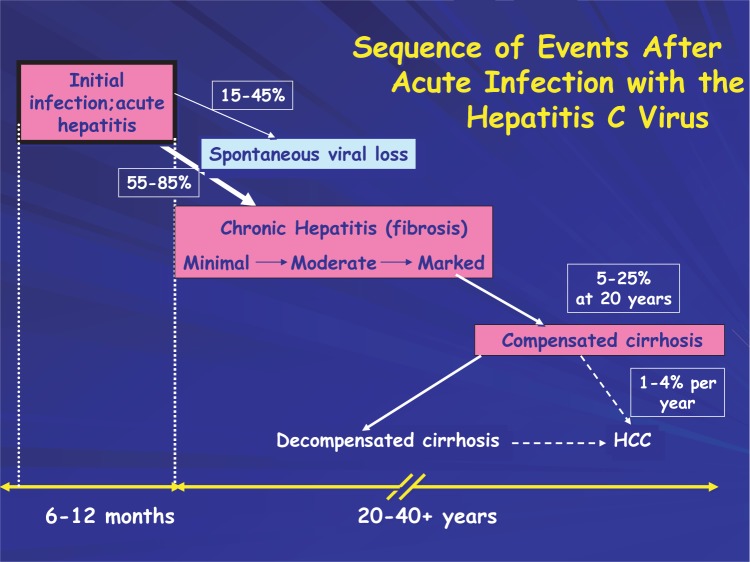
Diagram of the natural history of infection with HCV.

HCV infection is the most common chronic blood-borne infection in the United States. According the National Health and Nutrition Examination Survey (NHANES), more than four million Americans have been exposed to HCV and therefore exhibit antibodies to the virus. This is approximately four times the number infected with HIV. A diagram of the natural history of chronic HCV is presented in Figure 5. Infection with HCV typically leads to chronic viremia—the existence of viruses in the bloodstream. A recent review of studies of HCV infection indicated that spontaneous clearance of virus occurs in approximately one in four individuals with at least six-months of medical followup after acute HCV infection (Micallef et al. 2006). Spontaneous clearance of HCV infection, or self-cure, is an area of intense research investigation. Recent studies have shown that a competent immune response, comprising neutralizing antibodies and cellular immune responses (CD4 T cells), in acute HCV infection (the first six months after initial viral exposure) is necessary for self-cure (Kaplan et al. 2006; Pestka et al. 2007; Ruys et al. 2008).

NHANES estimates that an estimated 3.1 million persons in the United States have active chronic HCV infection (Table 1). The peak prevalence at the time of NHANES III (conducted from, 1988 to1994) was in persons 30 to 49 years of age (Alter et al. 1999), and more recent NHANES (1999–2002) shows, as expected, the highest prevalence of chronic infection is now in persons 40–59 years of age. The health care costs of illness and death associated with HCV infection in the United States is estimated to be $5.46 billion annually (Leigh et al. 2001).

Alone or in combination with alcohol consumption, HCV infections account for about 60 percent of all newly diagnosed cases of chronic liver disease and are the leading reason for liver transplantation as well as a major cause of liver cancer in U.S. residents (Chitturi and George, 2000; NIDA, 2000). Overall, HCV is responsible for up to 70 percent of chronic hepatitis cases, 30 percent to 40 percent of cases of cirrhosis and end stage liver disease, and 60 percent of liver cancer cases (CDC, 1998).

Injection drug use is the major high-risk activity associated with HCV infection. Through the implementation of HIV prevention interventions during the late 1980s and early 1990s, and subsequently through HCV education, the spread of HCV infection within the injection drug use risk group has slowed. Prospective studies have shown that 55 percent to 85 percent of exposed persons will develop a chronic infection with the virus, and up to 50 percent of patients, including injection drug users, may clear the virus (spontaneous self-cure) during acute infection (Jauncey et al. 2004). Estimates of self-cures of HCV infection in drug users vary greatly due to a number of parameters, including the difficulty of identifying acute HCV and accessing care for drug users (Amin et al. 2007). In a study of viral clearance in drug-abusing veterans, increasing age at the time of HCV infection, alcohol consumption, and HIV co-infection were associated with decreased likelihood of spontaneous HCV infection clearance (Piasecki et al. 2004). Another study showed the feasibility of using a prison setting or entry into detoxification as an environment in which to successfully identify acute HCV infection and treat those that do not self-cure (McGovern et al. 2006).

Persons who do not clear HCV infection may develop progressive liver disease and HCV-induced cirrhosis, which occurs in up to 20 percent of persons after roughly 20 years of chronic infection (Fig. 5). Approximately one-quarter of persons progressing to cirrhosis may develop end-stage liver disease and become candidates for liver transplantation. Patients who develop decompensated cirrhosis have a high likelihood of dying from complications of liver disease. Currently, an estimated 8,000 to 10,000 persons in the United States die from liver disease as a result of HCV infection each year, and the CDC has predicted that HCV-related mortality could triple over the next two decades (CDC, 1998).

Preventing the development of additional comorbidities by vaccinating against HAV and HBV infections as well as referring for substance abuse treatment are fundamental to the medical management of chronic HCV. For injection drug users, this can best be achieved by integrating prevention, care, and treatment for both substance abuse and HCV infection (Edlin et al. 2005).

National reporting data indicate that injection drug use accounts for the majority of reported acute HCV infections, greatly exceeding all other transmission factors (CDC, 2003a). Sexual exposure is the next highest risk factor accounting for up to 30 percent of cases of HCV infection, and transmission by this route is associated with multiple sexual partners and other sexually transmitted diseases. A recent study of the HCV incidence in a population of men having sex with men showed high incidence of HCV infection associated with HIV infection and ulcerative sexually transmitted diseases and rough sexual techniques (van de Laar et al. 2007). These data suggest a men having sex with men HCV transmission network. Almost all blood transfusion-related cases occurred prior to initiation of blood product screening in 1992. Other transmission routes include health care-related cases (e.g. accidental needle-stick or unclean medical procedure equipment), hemodialysis, non-sterile tattooing, and mother-to-child transmission during birth. In a small but significant number of cases, the etiology cannot be identified (Alter et al. 1999; CDC, 1998).

#### Treatment of Hepatitis C virus infection

The medical management of HCV has been addressed by consensus statements or clinical practice guideline development groups in the United States (AASLD, 2004; NIH, 2002), Canada (Sherman et al. 1997), France (Galmiche, 1998), and Europe (EASL, 1999). According to current HCV treatment guidelines, all patients with chronic HCV infection are potential candidates for antiviral therapy. Drug users, individuals with a history of drug use, or individuals in drug addiction treatment should not be excluded from needed HCV treatment as a result of drug use issues (AASLD, 2004; Scott, 2005). The latest update of treatment guidelines from NIH does not specify the need for a drug-abstinence period but indicates that patients should participate in drug treatment as an important adjunct to HCV therapy. Generally, patients with biopsy-proven liver disease who are at increased risk for progression to cirrhosis and end-stage liver disease are considered to be treatment candidates. As shown in Table 2, there are factors that influence the outcome of treatment for hepatitis C infection. In addition, those with factors associated with increased risk of rapidly progressive liver disease, such as HIV/HCV or HBV/HCV co-infection, are also candidates for treatment. A large retrospective Veterans Administration study has shown that individuals who are diagnosed with a substance use disorder (SUD) complete and respond to interferon-based HCV treatment regimens at similar rates to veterans without SUD's (Huckans et al. 2007). Because individuals, including injection drug users, with acute HCV infection may be highly responsive to interferon therapy, consideration should be given to early treatment during acute HCV infection (Calleri et al. 2007; Corey et al. 2006). A short course (12 weeks) of pegylated interferon-alfa treatment has been shown to be effective for injection drug users diagnosed with acute HCV infection (Calleri et al. 2007; DeRosa et al. 2007). The criteria for acute HCV infection in theses studies were one of the following: a) HCV antibody sero-conversion in the past 6 months; b) first at-risk exposure to HCV in the past 6 months; or c) elevated liver enzyme levels in the year prior to infection (normal liver enzyme level prior to infection. Treatment response times of as early as four weeks after the initiation of treatment appear to correlate with successful treatment outcomes (Bryan, 2007). Individuals with cirrhosis can be offered pharmacotherapy for HCV. However, those with signs of hepatic decompensation (such as ascites, persistent jaundice, wasting, variceal hemorrhage, or hepatic encephalopathy) are at high risk for treatment-related complications and death and should be referred for clinical trials or liver transplantation.

New pharmacologic agents and combination treatments introduced during the past decade have made treatment of chronic HCV infection increasingly effective. Sustained virologic response (SVR) is the benchmark of treatment success; it is defined as an undetectable viral load 6 months after the end of treatment. Approximately 55 percent of uncomplicated patients treated with current antiviral regimens can expect a sustained virological response. Follow-up studies of these patients show that nearly all have remained free of the virus (Kjaergard et al. 2001; Lang, 2007). Responses as high as 90 percent have been achieved in select populations. However, the development of similarly effective treatment options for patient groups at high risk for treatment-related complications and progressive liver failure remains an ongoing challenge (Davis and Rodrigue, 2001; Manns and Wedemeyer, 2001).

To date, the standard therapy for chronic HCV infection is the combination of pegylated (long acting) interferon and ribavirin; this combination has improved overall sustained virological response to greater than 50 percent (Davis and Rodrigue, 2001). Maximium sustained virological responses may occur in treatment with pegylated interferon and weight-based ribavirin (Torriani et al. 2004). Induction regimens, lengthier treatment regimens, consensus interferon, albumin interferon, and gamma interferon have all shown efficacy in preliminary trials. The use of mycophenolate mofetil and amantadine as adjunctive agents is also under study.

Reports at the American Association for the Study of Liver Disease, 2007 meeting indicated the greatest potential for new treatment breakthrough lies in orally available small molecules that target the HCV protease or polymerase (Sigal and Jacobson, 2007). Phase II clinical trials of HCV protease inhibitors and HCV polymerase inhibitors are revealing rapid declines in HCV levels (Afdhal et al. 2004; Reesink et al. 2006; Chu et al. 2004). However, due to the generation of resistant viruses, the antiviral therapies are provided in combination with current interferon-based treatment regimens (Lang, 2007a; Sarrazin et al. 2007). Other potential therapies are in development, such as synthetic oligodeoxynucleotides, and it will be several years before the efficacy of these newer products can be determined through clinical trials and become standard treatment regimens (McHutchison et al. 2007).

### Hepatitis C treatment: Complementary and alternative medicine

Approximately one-third of patients with chronic liver disease have been reported to use complementary and alternative medicines, and many use them without consulting their physicians (Seeff et al. 2001). NIH's National Center for Complementary and Alternative Medicine (NCCAM) is careful to note that “no complementary medicine or alternative medicine therapies have been scientifically proven to cure or even ease symptoms of hepatitis C” (NCCAM, 2000, p. 2).

Silymarin (milk thistle) is the complementary medication most frequently used, but St. John's wort, ginkgo biloba, ginseng, garlic extract, echinacea, and “Liverite” (a liver hydrolysate containing amino acids, vitamin B_12_, choline, inositol, lecithin, phosphatidylethanolamine, and phosphatidylinositol) are also commonly taken in an attempt to minimize the liver damage caused by HCV infection (Modi et al. 2007; NCCAM, 2000;). Milk thistle extracts have been shown to be have anti-inflammatory and anti-viral properties in addition to being well tolerated with minimal adverse effects (Polyak et al. 2007; Tamayo and Diamond, 2007). However, the interactions of these agents with interferon-based treatment regimens and adjunctive medications (e.g. methadone, antidepressants, etc.) are not known, but such “drug-drug interactions” may be significant, and certain alternative medications such as kava-kava have been associated with the development of fulminant liver failure.

### Hepatitis and HIV co-infection

Viral hepatitis and HIV infections are intersecting epidemics among injection drug users and possess many shared public health and treatment concerns (Bonacini, 2002; Peters, 2005). One survey of 295 patients entering an Opioid Treatment Program (OTP) found a prevalence of markers for HCV, HBV, and HIV of 80, 65, and 32 percent, respectively. Among the HIV-positive patients, 88 percent also were positive for HCV or HBV exposure (Chamot et al. 1992). Thus, viral hepatitis and HIV co-infection may be common among patients seeking or receiving treatment for opioid dependence.

#### HBV-HIV co-infection

Among patients infected with HIV, rates of chronic HBV infection range from 7 to 10 percent, with 80 percent of patients showing evidence of past or current HBV infection (Osborn et al. 2007). In injection drug use cohorts rates approaching 70 percent have been reported (Shire and Sherman, 2005). A study of HBV and HIV transmission has shown that HBV is sexually transmitted nearly nine times more efficiently than HIV (Kingsley et al. 1990). Therefore, sexual transmission of HBV and the intravenous inoculation of HBV through injection drug use need to be considered as potential transmission routes.

HIV infection modifies the natural history of HBV infection. Individuals with HIV infection are less likely to spontaneously clear or resolve HBV infection and therefore more likely to become chronic carriers of HBV. The ability to spontaneously clear HBV infection is dependent on generating an immune response to infection. For individuals infected with HIV, immune competence is a function of their CD4 count. Thus, managing HIV infection and maintaining elevated CD counts can be keys to managing the early stages of HIV/HBV co-infection. However, HIV induced immunodeficiency can reduce the immune mediated liver disease induced by HBV infection, but promote HBV replication. Reconstituting an immune response in HIV/HBV chronically co-infected patients through the use of antiretroviral therapy may result in enhanced liver damage and an initial flare up in liver enzymes. Studies of HBV/HIV infected patients show higher rates of liver-related mortality as well as increased progression of HIV infection (Konopnicki et al. 2005; Shen et al. 2004; Thio et al. 2002).

### Treatment of HBV-HIV co-infection

Advances in antiretroviral therapy have prompted a renewed interest in the medical management of HBV/HIV co-infection (Alberti et al. 2005; Nunez and Soriano, 2005; Peters, 2005; Shire and Sherman, 2005; Soriano et al. 2005). For co-infected patients, control of HIV infection is the priority. With the control of HIV, patients who are candidates for HBV therapy have the same treatment goals as individuals infected with HBV alone. Although there are currently no FDA approved drugs for the treatment of HBV/HIV co-infection, pharmacotherapy options include interferon-a (pegylated), lamivudine, adefovir, tenofovir, emtricitabine, and entecavir. The multiple antiviral options available allow for combination regimens and salvage therapy once drug resistant virus develops.

Table 3 provides representative treatment options targeting specific aspects of co-infection for patients with HBV/HIV co-infection based on either the U.S. Public Health Service Treatment Guidelines (Benson et al. 2004), the Spanish Consensus Conference recommendations (Soriano et al. 2004), the European Consensus Conference Guidelines (Alberti et al. 2005) or the recommendations of an International Panel of Experts (Soriano et al. 2005). The various treatment options, available guidelines, and treatment challenges have resulted in a variety of treatment practices for the management of HIV/HBV coinfection (Gaglio et al. 2006).

#### HCV-HIV co-infection

**Figure 6:**
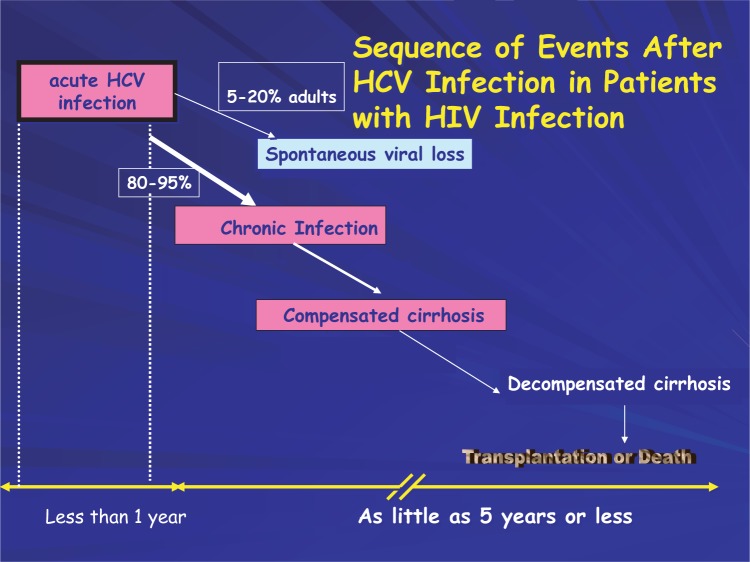
Diagram of the natural history of HCV infection in patients with HIV infection.

Eighty percent or more of injection drug users infected with HIV also test positive for exposure to HCV. The mode of transmission is through sharing of injection equipment resulting in the intravenous inoculation of virus. The majority (80 percent to 85 percent) of those exposed to HCV will become chronically infected (Fig. 6).

Most research studies indicate that HCV-positive persons co-infected with HIV tend to have more rapid declines in health, even when they receive antiretroviral therapy for HIV infection (Alvarez and Latorre, 2004; Greub et al. 2000; Sulkowski et al. 2007). HIV co-infection has also been shown to shorten the survival time of patients with HCV-related decompensated cirrhosis (Pineda et al. 2005). An investigation has shown that in a population of patients, HCV co-infection did not alter some health parameters: the risk of dying, developing acquired immune deficiency syndrome, or responding immunologically to antiretroviral therapy (Sulkowski et al. 2002). Prior to implementation of antiretroviral therapy, life expectancies were shorter and progressive liver disease was less evident in co-infected injection drug users. In the antiretroviral therapy era, life spans of patients with HIV infection are increasing, and end-stage liver disease is emerging as a major cause of morbidity and mortality in this population.

### Treatment of HCV-HIV co-infection

There is growing experience with treating HCV infection in HIV co-infected persons (Dore and Thomas, 2005; Mauss and Rockstroh, 2005; Mehta et al. 2006; Soriano et al. 2007). The medical management of patients infected with HIV and HCV remains a significant medical problem (Mehta et al. 2006). Medical management and treatment recommendations for HCV infection in HIV-infected individuals are available from the Hepatitis C Resource Centers (Department of Veterans Affairs, 2005), the Health Resources and Services Administration, HIV/AIDS Bureau (Swan, 2006), and a HCV-HIV international panel (Soriano et al. 2007). HCV-related liver disease in patients with HCV/HIV co-infection is a significant medical management issue. Thus, treatment guidelines for the management of HCV recommend that patients with HIV/HCV undergo medical evaluation for HCV-related liver disease. Liver biopsy remains the gold standard for the evaluation of liver disease (Sterling, 2005), but efforts are underway to develop noninvasive surrogate markers to accurately stage mild versus advanced liver disease in patients with HIV/HCV co-infection (Kelleher et al. 2005).

The level of liver disease is a consideration for HCV treatment (Aranzabal et al. 2005). Treatment of patients with HIV/HCV co-infection is further complicated by the relatively high prevalence of other medical and psychiatric comorbidities as well as the influence of each infection on the other. Compared with infection only with HCV, HCV/HIV co-infection results in a shorter interval for the appearance of clinically relevant liver disease, accelerated progression of liver disease, and increased mortality as a result of HCV-induced liver disease (Fig. 6). The treatment of HIV with antiretroviral regimens may result in an increase of HCV viral load and liver toxicity. Individuals, who develop a hypersensitivity to nevirapine during the course of treatment for HIV, have a sevenfold increase in their risk of death (Phillips et al. 2007). Other HIV treatment issues involve coin-fected patients receiving pharmacotherapies as part of their substance abuse treatment plan. They include substantial drug-drug interaction between methadone and antiretroviral medications as well as pharmacokinetic interactions between buprenorphine and efavirenz (McCance-Katz, 2005). Elevated HCV viral loads do not predict good outcomes for HCV treatment and are associated with post treatment relapse of coinfection (Nunez et al. 2007). Effective interferon-based treatment of HCV/HIV-infected individuals correlates with an early virologic response at 12 weeks of treatment (Montserrat et al. 2007); thus, allowing an early stoppage of treatment for those patients not responding to treatment.

A reduced treatment response, compared to HCV monoinfection, to HCV treatment in HCV/HIV populations is clearly evident from the data of three large-scale treatment trials (Carrat et al. 2004; Chung et al. 2004; Torriani et al. 2004). These three clinical trials reported similar sustained viro-logic response despite having diverse clinical trials designs. The APRICOT international clinical trial provided the best SVR to HCV treatment of HCV/HIV co-infected patients at 40 percent (Carrat et al. 2004). This study shows the importance of maximizing ribavirin concentration in combination with interferon treatment for patients with minimal liver disease to produce a SVR.

The NIH-supported AIDS Clinical Trials Group (ACTG5071) study reported a 27-percent SVR for co-infected patients with a history of drug use and minimal liver disease (Chung et al. 2004). This study reported a sustained virologic response of 15 percent for the patients infected with HCV genotype 1, the major HCV genotype observed in injection drug users.

The RIBAVIC study, a European study with a majority of injection drug users, reported a 27-percent SVR in patients who completed treatment, with a SVR for genotype 1 of less than 10 percent (Torriani et al. 2004). In this study, nearly half the patients were unable to complete the treatment regimen, a fact that underscores the difficulty facing health care providers in providing HCV treatment for co-infected individuals and the need for support services for these patients to maximize successful outcomes. Premature treatment discontinuation continues to be a prominent aspect of treatment clinical trials for HIV/HCV coinfected patients (Soriano et al. 2007a). Thus, HCV/HIV co-infected individuals who are in most need of effective treatment regimens find treatment difficult to complete and are least likely to respond to interferon-based treatment regimens.

Efficacy of treatment of acute HCV infection in patients with HIV infection has been shown in a pilot study (Dominquez et al. 2006). In this study, a 71-percent sustained virologic response was obtained in patients who were treated with peginterferon alfa-2a who had detectable HCV RNA 12 weeks after diagnosis. Thus, in this study, early treatment of acute HCV infection was highly successful.

An important measure in the response to treatment in HCV/HIV co-infected patients may be immune competence (Graham et al. 2005). However, the hallmark of HIV infection is the gradual loss of CD4^+^ cells as the infection progresses from acute to chronic. Progression of liver disease in the immunocompromised host is accelerated, but the immunopathogenic events that take place during this progression are poorly understood. The presence of CD4^+^ cells may be required for HCV clearance and self-limited disease (Post et al. 2004). A weak or limited CD4^+^ response to HCV antigens has been shown to be associated with a rapid progression of liver disease related to HCV infection, both in transplantation and nontransplantation settings. In patients with HCV/HIV co-infection, CD4^+^ T cell proliferative immune responses to HCV antigens are lower than in HCV monoinfected patients. Thus, in HCV/HIV co-infection, there may be a loss of recognition of HCV antigens and/or the loss of CD4^+^ helper function to induce CD8^+^ cytolytic cells which neutralize cells infected with HCV (Einav and Koziel, 2002). Immune enhancement strategies may be important in HCV/HIV co-infection to reduce the depletion of CD4^+^ cells thereby promoting both host defense mechanisms and enhanced responses to therapeutic regimens.

## Treatment of Viral Hepatitis Infection in the Context of Pharmacologic Therapy Provided in Opioid Treatment Programs

There are many types of substance abuse treatment programs that provide a variety of services for HIV/AIDS, hepatitis infection, and other sexually transmitted diseases (Brown et al. 2006). Opioid treatment programs (commonly referred to as OTPs or methadone programs) help individuals dependent on opioids abstain from illicit drug use through the dispensing of opiate agonist pharmacotherapies and other wrap around services. These programs range in the number of services provided and some programs provide a comprehensive therapeutic program that incorporates primary medical care, psychosocial counseling, vocational rehabilitation, HIV testing and counseling, viral hepatitis education and testing, and other vital medical and social services. Comprehensive “one stop shopping” integrated health service programs for injection drug users have been shown to promote good clinical treatment outcomes (Grebely et al. 2007; Sylvestre and Zweben, 2007).

Substance use disorders are complex chronic brain diseases with vast social costs that include crime, poverty, and devastating impacts on individuals and families (Brown, 2004) and “one stop shopping” integrated health service program can impact the ability of injection drug users to address social issues. Injection drug use—common among men and women who abuse or are dependent on heroin, cocaine, methamphetamine, and prescription opioids—often involves shared needles, non-sterile conditions, and other high-risk behaviors that can result complex health problems and require substantial care and services for the medical management of the consequences of substance abuse or dependence (Table 4).

Drug or alcohol abuse/dependence can cause direct damage to the liver, and substance-induced liver disease may be compounded by infection with viruses which may result in liver disease that requires medical care and is difficult to treat. The Centers for Disease Control and Prevention (CDC) current hepatitis C fact sheet indicated that most cases of HCV infection are due to injection drug use while the National Prevention Plan indicated that 60%–80% of persons who have injected drugs for at least 5 years are infected with HCV (CDC Viral Hepatitis C Fact Sheet; CDC National Prevention Plan).

Many patients dependent on opiates also abuse other drugs and may meet criteria for several substance use disorders. For example, patients dependent on heroin also have high rates of comorbid alcohol and/or cocaine abuse (Conway et al. 2003; Costenbader et al. 2007; Watson et al. 2007). For these individuals, the combination of medication, brief/behavioral interventions, and network therapy/peer support groups—which utilize family members and/or friends to support compliance with drug treatment (Galanter et al. 2004)—may be most useful to reduce risk-taking behavior and enhance their quality of life. Alcohol consumption exacerbates co-occurring liver disease, and patients with viral hepatitis infection should not consume alcohol (Kulig and Beresford, 2005). Alcohol is associated with elevated viral loads among patients infected with HCV, and the combination of elevated HCV loads and alcohol use is associated with a poorer therapeutic response to treatment (Finucane et al. 2007). Alcohol consumption reduces the survival rate among patients with hepatocellular carcinoma, a liver cancer that can result from chronic HBV or HCV infection (Wong et al. 2005).

### Care and treatment of hepatitis infection and opioid abuse and dependence

Prevention of and medical care for liver disease should be provided to patients in drug treatment and recovery programs in a comprehensive fashion to promote positive medical outcomes (Birkhead et al. 2007; van Beek, 2007). Enrollment in primary care and patient education about liver disease and prevention of infectious diseases are important in the medical management of viral liver infections (Edlin et al. 2005). Counseling/education of young injection drug users about prevention of infectious disease is particularly important. A recent study evaluating a behavioral intervention for young injection drug users comprising peer HIV and HCV education skills has shown that interventions that provide information, enhance risk-reduction skills and motivate behavior change can reduce injection risk behaviors (Garfein et al. 2007). Young injection drug users are seldom vaccinated to prevent viral hepatitis infection (Elefsiniotis et al. 2006; Kuo et al. 2004), even though vaccination may be the best course of action (Baral et al. 2007). Counseling individuals who already have hepatitis infection about a healthy lifestyle promotes treatment readiness among patients with progressive liver disease (Zweben, 2001). Treatment readiness interventions are important for injection drug users in their access to care and treatment. Injection drug users do not receive treatment for hepatitis C infection in great numbers (Grebely et al. 2008). Developing treatment readiness through patient education and counseling with the use of peer educators and support groups can reduce the number of patients refusing treatment for hepatitis infection (Moirand et al. 2007; Schackman et al. 2007; Sylvestre and Zweben, 2007). Providing integrated primary care and pharmacologic treatment for opioid dependence can facilitate both recovery from opioid dependence and medical treatment of co-occurring conditions, such as viral hepatitis infections (Fiellin et al. 2003; Litwin et al. 2007).

Current clinical practice guidelines recommend care and treatment for patients infected with viral hepatitis who might benefit from treatment and virus eradication. However, barriers seriously limit this care and treatment for injection drug users (Edlin et al. 2005; Grebely et al. 2008; Nguyen et al. 2007; NIH, 2002; Schaefer et al. 2004). One significant barrier is the need for more data on program structure and elements supporting effective HCV treatment for injection drug users. A review of the research clinical trials literature published between 1987 and 2004 and focusing on the treatment of chronic HCV infection describes only 10 clinical trials involving patients with drug abuse (Robaeys and Buntinx, 2005). None of the published clinical trials randomized patients and only one used pegylated interferon, the medication currently considered the standard of care for HCV treatment. Recently, more prospective, controlled clinical trials of standard-of-care treatments for HCV infection in patients who are injection drug users have been performed. In 2007, seven treatment trials were published that included injection drug users (Belfiori et al. 2007; Calleria et al. 2007; DeRosa, et al. 2007; Grebely et al. 2007; Huckans et al. 2007; Krook et al. 2007; Schaefer et al. 2007) while other studies investigated factors related to the successful treatment of injection drug users including drug-drug interactions with methadone (Berk et al. 2007; Gupta et al. 2007), immune responses of injection drug users (Sergi et al. 2007), and medication adherence (Sylvestre and Clements, 2007). More studies are needed to enhance the development of effective treatment programs and clinical guidelines.

There are numerous reasons for the lack of large clinical trials resulting in clinical guidelines for the medical management of co-occurring hepatitis infection and drug abuse/dependence. These include the generalized stigma and prejudice associated with substance-dependent persons, their disenfranchisement from the medical community, their complex medical management issues, health-care providers' lack of current treatment knowledge about patients who are injection drug users, the design of clinical trials to exclude injection drug user participation, as well as a lack of infrastructure to deliver effective care and treatment to injection drug users (Dore and Thomas, 2005; Edlin et al. 2001; Rauch et al. 2005). Thus, substance abusers are rarely able to meet the strict eligibility criteria established for entry into many studies using interferon therapy. Consequently, treatment for chronic liver disease may be delayed or withheld for current or former substance-dependent patients, as well as for those in recovery who are receiving treatment for drug abuse. Individuals with untreated hepatitis infection are at risk of progressing to end-stage liver disease or decompensated cirrhosis, leaving liver transplantation as the only life-saving alternative. More liver transplants are performed for HCV-related infection (30–46 percent of transplants) than for alcohol-related disorders (23–25 percent) (Botero, 2004; Mandayam et al. 2004).

### Patient/provider relationship and care and treatment for hepatitis infection

Care and treatment of hepatitis infection and other comorbidities associated with injection drug use is complex, and numerous barriers prevent high-quality care and positive medical outcomes. Patients vary over a wide range of engagement in care and treatment, as well as various stages of readiness to seek care. Some patients do not know whether or not they have hepatitis infection or other comorbidities. Others, with known hepatitis infection, may not have been referred for medical evaluation with care and treatment or did not follow though on the referral. Others are actively involved in treatment for hepatitis infection and other comorbidities.

Injection drug users and those who are at risk for viral hepatitis are more likely than the general population to suffer psychiatric disorders such as major depression, anxiety disorder, and bipolar disorder, and some patients use drugs or pharmaceuticals in an attempt to self-medicate an underlying psychological illness. Such untreated co-occurring disorders may increase risk-taking behaviors, and this scenario is further complicated by negative experiences of injection drug users with the health care system (Davis and Rodrigue, 2001; Golub et al. 2004; Stein et al. 2003).

A trusting relationship with a member of the healthcare team who can help patients anticipate, plan for, and endure the difficulties that arise in the medical management of drug abuse/dependence and its associated comorbidities is fundamental for drug users who seek care. This engaging relationship can be facilitated by peer support groups (Sylvestre and Zweben, 2007). A patient-provider relationship that will support a dialogue in which both parties are able to communicate openly about their expectations and frustrations is critical. However, the health care system may not support such a dialogue (Shine, 1996). Drug users often believe that the health care they receive is judgmental and condescending, unresponsive to their needs, and often delivered without respect. As a result, drug users may display individual barriers to accepting therapy and fail to follow through with medical advice, or take prescribed medication, or keep appointments (Mehta et al. 2005).

The extensive experience gained from treating injection drug users for medical conditions, especially HIV infection, has led to the development of effective principles for engaging drug users in health care relationships. Successful programs have a respectful approach to drug users, understand the medical and behavioral aspects and consequences of drug abuse and dependence, refrain from moral judgments and utilize a multidisciplinary team approach (Batki and Sorensen, 1999; O'Connor et al. 1994; Robertson, 1998; van Beek, 2007). These strategies embody a client-centered approach in which a care provider works with a client to identify changes that the client is motivated to make to enhance health and well-being (Brands et al. 2003). Even if global behavior change (such as ceasing all drug use) is not possible, other measures can reduce the medical consequences of high-risk behavior (Des Jarlais et al. 1993). In this setting, health care providers can work with the patient to develop a care and treatment regimen that is able to fit the lifestyle of the patient (e.g. once-daily therapy) rather than attempting to restructure the patient's lifestyle.

Misunderstandings about the nature of substance use disorders as chronic, potentially recurring diseases influence the nature of the relationship between the patient and the provider. Relapse during care often is perceived as failure in drug treatment rather than a characteristic of the disease. In fact, relapse may actually be relatively benign, with brief “lapses” of sobriety or abstinence sometimes called “slips” (Finney et al. 1999; Graham et al. 2002). In the context of ongoing substance abuse treatment, neither lapses nor relapses represent permanent barriers to recovery.

Strong patient-provider relationships are essential, because treatment regimens for chronic HBV and HCV infections are difficult and stressful for patients. Drug users should be presented with a comprehensive health program that incorporates high-quality hepatitis prevention and care, and substance abuse treatment. Hepatitis prevention and care should include outreach to drug users through peer educators/support groups, screening and counseling for at-risk behavior, HCV infection testing and genotyping, HBV infection testing, HIV infection testing, prevention counseling and hepatitis education, vaccination against HAV and HBV infections (if eligible), and evaluation for comorbidities (Edlin et al. 2005). This evaluation should include determining the need for substance abuse services, psychiatric care, and social support. It also should include an effort to engage the patient in primary care, as well as a liver evaluation and an assessment for treatment of chronic HBV infection and/or HCV infection. Treatment trials of chronic HCV infection have shown that programs that employ a multidisciplinary team and address co-occurring psychological disorders result in excellent treatment outcomes compared with programs that do not address treatment barriers (Bargiacchi et al. 2005; Broers et al. 2005; Sylvestre and Zweben, 2007).

It is possible—and important—to prevent progression to injection drug use by encouraging individuals who abuse noninjection drugs to enter treatment. Specific risk factors appear to lead individuals to make the transition to injection drug use. For example, individuals who engage in inhalant drug use in early teenage years tend to have a higher likelihood of progressing to injection drug use. In addition, certain factors appear to influence whether those who inhale drugs progress to injection drug use: Those who inhale drugs and who also have an intact family structure appear less likely to progress to injection drug use; homeless individuals and those whose sex partners inject drugs are more likely to progress to injection drug use (Lankenau and Clatts, 2004; Maxwell et al. 2004; Storr et al. 2005).

The importance of preventing individuals from progressing to injection drug use can be vividly seen in data comparing the HCV infection incidence among injection and noninjection drug users (Fuller et al. 2004). This longitudinal surveillance study in New York City showed an annual incidence rate of HCV infection in young noninjectors of 0.4/100 person years, compared with 35.9/100 person years for injection drug users. Delaying or preventing the transition to injection drug use may have a significant health benefit by reducing the risk of comorbid conditions associated with injection drug use and drug abuse/dependence.

### A comprehensive substance abuse treatment plan and pharmacologic therapy

Substance abuse is a complex physiologic, social, and behavioral disorder that often coexists with psychiatric illness as well as comorbid medical conditions. For this reason, screening substance users for comorbid psychiatric illness should be considered an integral part of any medical intervention and comprehensive substance abuse treatment program (Sylvestre et al. 2004). It may be difficult to determine which comorbidity—substance abuse, mental illness, or infectious disease—should be addressed first. However, medical treatment of substance-related disorders often is necessary to create sufficient stability to begin treatment of other conditions. Stability is further increased when mental health services and substance abuse treatment are combined, enhancing the medical outcomes of treatment for comorbidities. *Substance Abuse Treatment for Persons with Co-Occurring Disorders*, Treatment Improvement Protocol (TIP) 42, (SAMHSA, 2005a) provides up-to-date information about co-occurring substance use and mental disorders as well as recommended best practices in the treatment of these disorders.

Understanding that substance abuse is a complex multifactor disorder, it is appropriate to develop, through case management, a comprehensive substance abuse treatment plan that comprises behavioral, social rehabilitative components, and biological (pharmacological) treatments (Table 5).

Pharmacological treatments have been developed and approved for specific drug addictions. Currently, addiction treatment medications are available for nicotine, alcohol, and opiates. Medications are now being developed for dependence and abuse of stimulants, like cocaine and methamphetamine. Several marketed medications—disulfiram, baclofen, modafinil, naltrexone, ondansetron, tiagabine, and topiramate—have shown efficacy to reduce cocaine use in initial clinical trials (Vocci and Ling, 2005). To date, no medications tested in clinical trials have shown efficacy to reduce methamphetamine use.

**Figure 7:**
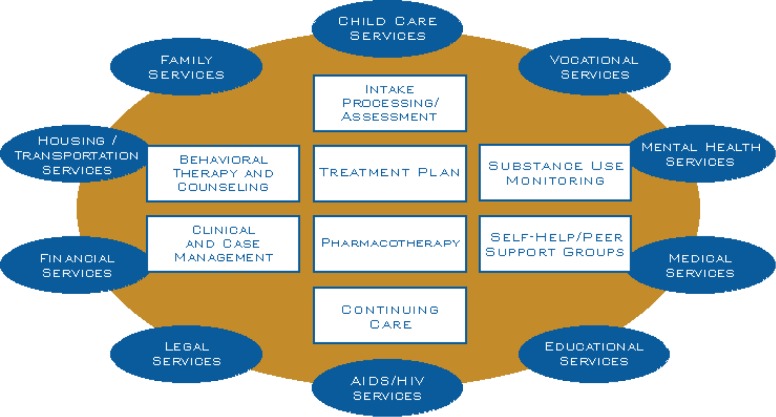
Diagram of the elements of a comprehensive substance abuse treatment plan (from NIDA 2000a).

Medications are a proven component of comprehensive substance abuse treatment plans that reduce drug use and provide an opportunity for improvement in health and social functioning for individuals with opioid dependence (Fig. 7; Gowing et al. 2004; Johnson and McCaugh, 2000; NIDA, 2000a). Two recent TIPs from SAMHSA, TIP 40 and TIP 43, provide the best practices guidelines for the use of either methadone or buprenorphine as part of a comprehensive treatment plan for opioid abuse/dependence (SAMHSA, 2004; SAMHSA, 2005).

Treatment services for drug abuse/dependence that follow recommended best medical practices are more likely to manage the care and treatment of hepatitis successfully and to prevent progressive liver disease. The medications used in the management of opiate dependence are metabolized through the liver, and therapeutic blood levels can be affected by liver disease. Two pharmacologic therapies, methadone and buprenorphine, illustrate the interaction between appropriate pharmacotherapy and the possible impact on liver disease.

#### Methadone

Methadone is the mainstay of pharmacotherapy treatment for opioid dependence and helps dependent individuals abstain from illicit drug use and achieve recovery. Methadone is a synthetic mu-opioid receptor agonist with pharmacological properties qualitatively similar to morphine. Administered daily as an oral dose, methadone should be present in the blood at levels sufficient to eliminate symptoms of opioid dependence during a 24-hour period, without episodes of opioid overmedication or withdrawal (Payte and Zweben, 1998). The blood level and elimination of methadone may be influenced by factors such as poor absorption, variable metabolism, other medications, diet, physical condition, patient age or pregnancy, and vitamins or herbal products such as St. John's wort. Therefore, considerable flexibility in dosing is required to stabilize patients and an adequate physiologic methadone level is critical for therapeutic success (Eap et al. 2002).

Methadone is safe when used as indicated (SAMHSA, 2005). Research studies have not demonstrated liver toxicity in patients with underlying liver disease. Serious adverse reactions or cumulative organ damage has not been reported when daily methadone is used in appropriate dosages. Mortality rates of patients in methadone treatment from all causes are typically one-third those of untreated opioid addicts (SAMHSA, 2004a). However, fatal overdoses with methadone, as well as deaths of clients in methadone treatment, have been reported (Clarck et al. 1995; Maxwell et al. 2005; Shah et al. 2005; Fugelstad et al. 2007). Data from Stockholm, Sweden show that patients receiving methadone treatment had a lower mortality rate and that leaving methadone treatment resulted in a 20 times increase risk in death due to drug overdose (Fugelstad et al. 2007). A study of patient deaths in methadone treatment in Texas (Maxwell et al. 2005) revealed 20 percent of deaths due to liver disease, 18 percent of deaths due to cardiovascular disease, and 14 percent due to drug overdose or trauma. In New Mexico, (Shah et al. 2005), 50.3 percent of deaths of patients in treatment between 1998 and 2002 were from methadone in combination with illicit drugs, 23.8 percent were from methadone in combination with prescription drugs (possible pain management patients), and 3.5 percent due to methadone in combination with alcohol. These data show the importance of other addictive drugs in combination with methadone in unintentional methadone-related deaths. In treatment, methadone-associated deaths can occur during the induction phase when a patient's level of tolerance to opioids is not correctly assessed or when a patient continues to use other central nervous system depressant drugs in combination with methadone.

#### Buprenorphine

Buprenorphine is a partial muopioid receptor agonist (Ling and Smith, 2002; see Table 5). At higher doses, buprenorphine reaches a plateau in its similarity to opioid properties. This limitation on agonist effects results in an improved safety profile compared with a full agonist such as methadone. Specifically, buprenorphine has a favorable “ceiling effect” on respiratory depression (Walsh at al. 1994). In addition to improved safety, flexible dosing (e.g. thrice weekly) is feasible since buprenorphine has a high binding affinity for the opiate receptor and dissociates slowly.

A report from France has noted an elevation in measures of abnormal liver function after the use of intravenous buprenorphine. This report was limited by the small sample size, retrospective analysis, and short time in which buprenorphine was given (Petry et al. 2002). However, the use of buprenorphine in individuals with known liver disease is of concern and many clinicians have avoided buprenorphine in this patient population. Since, 2001, no additional reports of liver toxicity with buprenorphine have been reported, despite increasing use of buprenorphine in treating opioid dependence. In 2002, the Food and Drug Administration (FDA) approved two sublingual buprenorphine products for use in the United States as a treatment for opioid dependence, and large-scale use of buprenorphine continues in Europe.

Buprenorphine may be a component of the substance abuse treatment plan for individuals infected with HCV (Alford et al. 2007; Belfiori et al. 2007; Bruce and Altice, 2007; Krook et al. 2007). Among patients treated through a mobile outreach intervention in New Haven, Connecticut, 36 individuals infected with HCV and HIV have been treated with buprenorphine. Liver function measures show no adverse effect from buprenorphine treatment in this co-infected population (Kresina et al. 2005). In a recent study of co-infected homeless opioid-dependent individuals, buprenorphine treatment was effectively implemented with comparable outcomes to housed patients treated with buprenorphine (Alford et al. 2007). Although monitoring is required when any medication is added to a patient's medication regimen, the presence of HIV/HCV co-infection or use of antiretroviral therapy does not rule out use of buprenorphine.

### Pharmacotherapy for Alcohol Dependence

Patients with co-occurring injection drug use, alcoholism, and liver disease may need treatment aimed at ending alcohol use. Medications for alcohol dependence include acamprosate, naltrexone (vivitrex), or disulfiram (Fiellin et al. 2004). Acamprosate and naltrexone have different mechanisms of action and modify different behavioral aspects of dependence. Acamprosate, a long-acting compound, prolongs periods of abstinence by normalizing glutamate neurotransmission that is disrupted during chronic alcohol consumption and withdrawal. Naltrexone, a fast-acting opioid receptor antagonist with a long half-life, can reduce heavy drinking by decreasing alcohol's rewarding effects. Safety and effectiveness of treatment using both drugs for alcohol addiction have been shown in double blind studies (Littleton and Zieglgansberger, 2003). A long-acting formulation of naltrexone, which would allow treatment of alcohol dependence with a monthly injection, is now FDA approved (Garbutt et al. 2005; Johnson, 2006). Disulfiram is designed to help motivate patients to remain abstinent from alcohol through “vicarious aversive therapy”—the patient who has taken disulfiram and then ingests alcohol experiences a series of unpleasant allergic-like symptoms (e.g. flushing, headache, and vomiting). The drug works by blocking the oxidation of alcohol at the acetaldehyde stage in its metabolism. *Incorporating Alcohol Pharmacotherapies into Medical Practice*, Treatment Improvement Protocol (TIP) 48, (SAMHSA, 2007) provides up-to-date information about the use of medications currently approved for treating alcohol use disorders.

### Integration of addiction treatment with hepatitis prevention, screening and treatment

Individual OTPs provide a range of services and some programs provide a comprehensive blend of therapies—primary medical care, psychosocial counseling, vocational rehabilitation, HIV testing and counseling, HCV education and testing, and other vital medical and social services—needed to effectively treat substance abuse, dependence and its associated comorbidities. Substance abuse treatment programs that offer a broader array and greater frequency of services report longer time in treatment and improved treatment outcomes. Programs that respond to the severity of drug abuse during the first stages of drug treatment show positive treatment outcomes related to longer retention in treatment and patient satisfaction with treatment services (Hser et al. 2004). Entry of injection drug users into substance abuse treatment is facilitated by program outreach and case management as well as the patient characteristics of not being homeless, having less problems with alcohol consumption and advancing though the stages of behavior change (Corsi et al. 2007). Maximum retention time in methadone treatment is associated with comprehensive treatment and provision of frequent health services, as well as appropriate methadone dosing (Booth et al. 2004).

Comprehensive services for hepatitis infection include hepatitis prevention, care, and treatment. Elements of hepatitis prevention and care for drug users include screening for at-risk behavior; HAV, HBV, HCV, and HIV testing; prevention counseling and education; vaccination against HAV and HBV infections; and evaluation for comorbidities, including the need for substance abuse services, psychiatric care, social support, liver disease evaluation, and interferon-based HCV treatment.

Injection drug use can lead to HCV infection. A recent incident infection study showed that women, new injection initiates and injection drug users recruited through outreach are at increased risk for HCV infection (Maher et al. 2006). Prevalence estimates of HCV infection derived from surveys of patients in methadone treatment programs range from 72 percent to more than 90 percent (CDC, 1998; Inglesby, 1999; McCarthy and Flynn, 2001; NIDA, 2000; Stein et al. 2001), compared with 1.8 percent in the overall U.S. population (Alter et al. 1999; CDC, 1998). In one study of 306 OTP patients, 82 percent had not received prior HCV testing and 87 percent were infected with HCV (Stein et al. 2001). The CDC recommends routine HCV testing for individuals who have ever injected illegal drugs as part of a national strategy to identify HCV-infected individuals and to enter into care to prevent the consequences of their infection (CDC, 1998). In addition, testing for hepatitis infection and peer-driven counseling can change injection drug users' risky behaviors that increase the risk of transmitting HCV (Aitken et al. 2002; Garfein et al. 2007; Latka et al. 2007; Tucker et al. 2004).

HCV treatment studies demonstrate that roughly one in five current alcohol and/or drug abusers do not comply with HCV treatment monitoring or are lost to followup. Thus, a consequence of continued drug use may be an increased viral load and reduced response to treatment (Davis and Rodrigue, 2001; Sylvestre, 2002). However, patients with co-occurring HCV infection and substance use can complete interferon-based treatment with careful monitoring and aggressive intervention. HCV treatment providers who integrate early interventions for drug use and other comorbidities into their HCV treatment plan improve the likelihood of good outcomes. HCV patients can successfully be treated with interferon-based therapy even if they have histories (or current incidence) of significant substance use disorders (Dore and Thomas, 2005; Grebely et al. 2007; Hopwood and Treloar, 2007; Sylvestre et al. 2005).

In the past, patients receiving methadone have not been included in clinical studies of HCV treatments because methadone treatment has been considered a confounding factor in determining treatment efficacy, the OTP population has been viewed as atypical HCV patients, and researchers have feared that some former and current injection drug users would not adhere to treatment. Recently, however, a growing number of studies (Berk et al. 2007; Gupta et al. 2007; Mauss et al. 2004; Robaeys et al. 2006; Schaefer et al. 2003; Sergio et al. 2007; Sylvestre et al. 2005; Van Thiel et al. 2003; Verrando et al. 2005) have found that interferon-based treatment regimens are safe and effective for patients receiving methadone treatment, that dosing of interferon or ribavirin is not altered by methadone, and that patients who discontinue HCV therapy while receiving methadone do so early in the course of HCV treatment.

Patients receiving methadone treatment should not be withdrawn from methadone prior to HCV treatment, as continued methadone maintenance can be helpful in enhancing quality of life through stabilization during HCV treatment. However, additional research is needed to better understand the natural history of HCV infection in patients receiving pharmacotherapy for substance use. Recent studies have added to the growing body of evidence indicating that interferon-based therapy is effective in substance abuse treatment settings for patients receiving methadone or buprenorphine as part of their treatment for opioid dependence (Belfiori et al. 2007; Krook et al. 2007). Therefore, AASLD Clinical Practice Guidelines recommend that HCV treatment not be withheld from individuals seeking or receiving substance abuse treatment. (Strader et al. 2004).

### Early screening, testing, and treatment for HCV infection

The best methods for detecting HCV infection are to screen populations for a history of at-risk behaviors and to test individuals who have an identified risk behavior or factor for HCV exposure (AASLD, 2004). Injection drug use is the chief mode of HCV transmission in the United States; therefore, anyone with a history of injecting drugs should be tested for HCV infection (CDC, 1998). Regardless of substance abuse status, individuals with HCV infection should receive counseling, education, medical evaluation, care, and needed treatment (Alter et al. 2004). Early treatment studies have shown high sustained virologic response in patients treated within 3 months of testing positive for HCV infection or 8 weeks post-exposure, reporting a sustained virologic response of greater than 90 percent (Calleri et al. 2007; Corey et al. 2006; Jaeckel et al. 2001; Normura et al. 2004). Treating acute HCV infection expeditiously is likely to prevent complications, such as cirrhosis, and to be cost-effective (Santantonio, 2004).

Unfortunately, it is difficult to identify recent or acute HCV infection in opioid-addicted persons first entering substance abuse treatment programs because other health problems or barriers may be present (Chitturri and George, 2000; Leavitt, 2001; Sylvestre, 2002; Watson et al. 2007). Exposure to HCV is determined by the presence of serum antibody to HCV through use of an enzyme immunoassay. HCV infection is determined by identifying HCV virus in samples of blood serum using molecular tests such as polymerase chain reaction and/or transcription-mediated amplification (NIH, 2002). In a study of 493 patients exposed to HCV who were in opioid treatment, 77 percent were found to have HCV infection as determined by polymerase chain reaction analysis. The only statistically significant clinical feature distinguishing those with HCV infection from others was abnormal levels of the liver enzyme alanine aminotransferase (Sylvestre et al. 2005). Fewer than half (30–40 percent) of patients display symptoms prior to testing positive for HCV exposure; the initial marker for HCV exposure may not be present in symptomatic patients. For some patients, acute HCV infection occurs without any signs and symptoms, which may not be apparent until cirrhosis develops. Once end-stage liver disease develops, prospects for survival are limited (Wong et al. 2004). There are important benefits to starting treatment during early or acute stages of HCV infection, but an accurate evaluation using appropriate laboratory screening techniques is needed, as laboratory methods to identify early HCV infection are not readily available.

### Hepatitis education

Drug treatment programs can provide a variety of services including education of patients about hepatitis infection, but patients may not be aware or utilize such a service (Strauss et al. 2007). OTPs are more likely than are drug-free treatment programs to provide hepatitis education materials to patients and to educate most or all staff about hepatitis infection (Astone et al. 2003; Strauss et al. 2003). Education materials provided in OTPs are more comprehensive and encompass topics such as viral transmission, testing, treatment options, and HIV co-infection (Strauss et al. 2004). SAMHSA supports a hepatitis education program for OTPs, and the American Association for the Treatment of Opioid Dependence (AATOD) has developed a curriculum for hepatitis education and participated in its dissemination through The Hepatitis Education Training for Opioid Treatment Providers Program (www.AATOD.org/hepatitis. html).

Other hepatitis curricula, developed by Federal and State agencies, are available (see Table 6) and emphasize differing aspects of HCV infection. For example, the New York State Department of Health curriculum includes a 2-day workshop that emphasizes integrating HCV into substance abuse treatment settings; the Veterans Administration curriculum is vast and provides great detail regarding HCV and alcohol consumption; and the Health Resources and Services Administration has developed a curriculum as technical assistance for HIV care providers that treat patients with co-occurring HCV infection.

Peer-driven counseling and education using a brief behavioral intervention and peer-driven counseling coupled to testing for hepatitis infection can change risky behavior associated with the HCV transmission among injection drug users (Aitken et al. 2002; Garfein et al. 2007; Tucker et al. 2004). These education and counseling efforts are important components of a comprehensive substance abuse treatment plan for injection drug users because, when implemented, they increase the patient's knowledge of HCV and promote treatment readiness (Evans et al. 2005; Sylvestre and Zweben, 2007; Walley et al. 2005). However, not every substance abuse treatment program serving injection drug users provides these needed services (Brown et al. 2006; Vassilev et al. 2004).

### Clinical research: Hepatitis C treatment studies and pharmacotherapy

Available data indicate that HCV treatment outcomes of patients receiving methadone treatment or buprenorphine, as part of their treatment for opioid dependence, can be equivalent to those reported in studies of patient populations that exclude patients receiving methadone treatment. Schafer (2001) found that 50 percent of patients receiving methadone experienced a viral response to interferon-ribavirin treatment after 6 months, compared to 39 percent of patients in a control population. Schafer concluded that patient compliance and retention in therapy were critical factors and that an interdisciplinary OTP setting with adequate patient support facilitated safe and successful treatment.

Blechman et al. (1999) compared interferon therapy for chronic HCV infection in 26 patients receiving methadone treatment with a control group of 22 patients not receiving methadone. Disease severity, response to interferon, side effects, and treatment compliance were equivalent in both groups. The authors concluded that stable patients receiving methadone treatment should not be excluded from HCV treatment trials and are candidates for antiviral therapy as noted in current Clinical Practice Guidelines (AASLD, 2004).

Hagan and colleagues (1999) examined interferon therapy administered to 19 HCV-infected patients in an OTP. Of the 14 (74 percent) who completed the study, 79 percent had a treatment response at 3 months. Only two patients were discontinued because of medication nonadherence; two left the OTP; and one discontinued interferon. This study showed that delivering interferon-based therapy in the OTP clinic setting is a feasible option.

**Figure 8:**
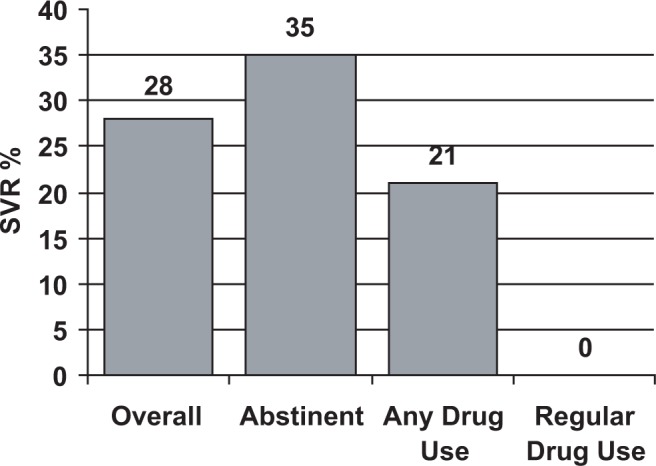
Sustained virologic response (SVR) to interferon-ribavirin treatment in 66 HCV-positive patients receiving methadone treatment (Sylvestre et al. 2005).

A study of HCV treatment in 76 recovering heroin users maintained on methadone found that neither current drug use nor short duration of abstinence before treatment led to significant reductions in outcomes for patients receiving standard regimens of interferon plus ribavirin (Fig. 8). The authors concluded that injection drug users can be safely and effectively treated for HCV despite multiple barriers to treatment when they are treated in a setting that can address their special needs (Sylvestre et al. 2005).

**Figure 9:**
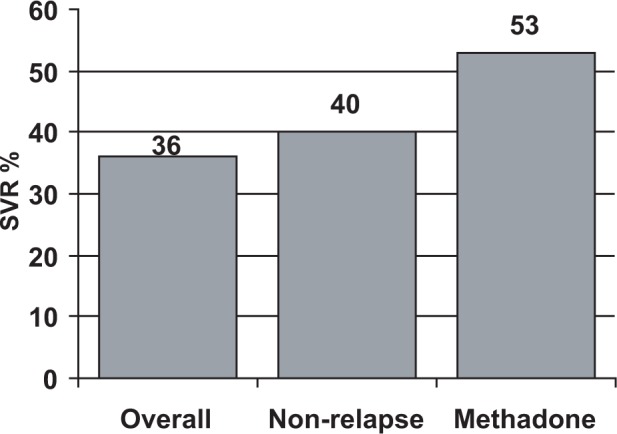
Sustained virologic response (SVR) among HCV-positive injection drug users (data from Backmund et al. 2001).

Another study investigated whether patients still injecting opioids and infected with HCV could be detoxified from opioids and successfully treated with interferon-ribavirin combination therapy. Fifty injection drug users, including a majority variously dependent on alcohol, cocaine, and/or benzodiazepines, underwent a 28-day opioid withdrawal program and received treatment for HCV infection (Backmund et al. 2001). Overall, there was a sustained virologic response in 36 percent of subjects who exhibited excellent medication compliance and clinic attendance. However, 80 percent of patients who had completed 28-day detoxification had one or more injection-drug relapses, including 10 of the 18 patients with a sustained virologic response. Thirty percent of the patients who relapsed had entered treatment at an OTP, and 53 percent of these patients receiving methadone treatment had a sustained virologic response. This response was higher than the rate in the overall group (53 percent versus 17 percent) and the rate in patients who remained abstinent without a drug relapse (53 percent versus 40 percent) who did not enter an OTP (Fig. 9). While substantial, these differences did not reach statistical significance, possibly because of the small numbers of subjects involved and the low statistical power.

Although this study indicates that injection drug users may be treated successfully for HCV infection in the face of continuing drug abuse, the researchers emphasize that this group of subjects was younger and had a relatively shorter duration of HCV infection than in other comparative studies. The study reported high relapse rates with no HCV re-infections. This may be due, in part, to subjects using the sterile syringes and needles provided for home injection of interferon. Other reports suggest that re-infection may not be as rapid as previously thought. In a study of 27 former injection drug users not in an OTP who had been treated successfully for HCV infection, one-third returned to injecting drugs and only one was found to be re-infected at 64-months followup. In another study that followed injection drug users for a mean of 33.8 months, 15 of 18 participants remained HCV RNA-negative (Dalgard et al. 2002; Backmund et al. 2004). Further studies are needed to clearly assess the risk of HCV re-infection in patients who relapse to injection drug use.

Stein and colleagues (2001) observed that the typical stability of persons in an OTP makes them good candidates for the rigors of interferon-based therapy. Of 306 HCV-positive patients in an OTP that they surveyed, 53 percent were eager to participate in interferon therapy even though they understood that interferon-based treatment requires injections, is only partially efficacious, may produce adverse reactions, and may require a liver biopsy.

Robaeys and colleagues (2006) showed in a retrospective cohort study of 406 patients with chronic HCV that noncompliance in injection drug users was not different from noninjection drug users. Moreover, the injection drug users group had a higher sustained virologic response (46.6 percent) than the noninjection drug users group (34.6 percent).

Two recent studies of patients receiving either methadone or buprenorphine in combination with psychosocial support showed effective treatment of HCV infection with peg interferon alfa 2a or 2b plus ribavirin (Belfiori et al. 2007; Krook et al. 2007). In the Norwegian study, treatment compliance was 100 percent, all patients responded to HCV treatment and 94 percent of patients obtained a sustained virological response (Krook et al. 2007). In the Italian study, three-fourths of patients were drug dependent for longer than 5 years prior to HCV treatment, 17 percent dropped out of treatment relapsing to drug use while 39 percent obtained a sustained virological response.

Thus, a growing body of evidence indicates that patients' receiving either methadone or buprenorphine, as part of their treatment for opioid dependence, can comply with interferon-based HCV treatment regiments with treatment outcomes producing a sustained virological response. In addition, patients receiving treatment at OTPs may exhibit good HCV treatment adherence and retention, with limited side effects or adverse events. AALSD Clinical Treatment Guidelines recommend that HCV treatment not be withheld from patients participating in substance abuse treatment programs. A period of abstinence from alcohol and illicit drugs may be beneficial for maximizing treatment responses but is not necessary or required to initiate interferon-based treatment of chronic HCV infection. Two critical factors in pharmacotherapy treatment for drug dependence and concurrent HCV treatment are (1) maintaining adequate methadone/buprenorphine dosages to avert potential drug-relapse both prior to, during, and following treatment for HCV infection and (2) conducting ongoing supportive psychosocial and psychotherapies for patients through the course of treatment.

### Viral hepatitis and the health care provider: Occupational and health care facility-related exposure to viral hepatitis

It is possible that HCV can be transmitted to patients being treated in a health care setting. In substance abuse treatment programs, both the health care provider and patient must be aware of this risk and of possible courses of action if viral transmission should occur.

The route of transmission for approximately 10 percent to 15 percent (Fig. 7) of all hepatitis infections remains undetermined. Nosocomial infections (i.e. infections that are a result of treatment in a hospital or hospital-like setting) and patient-to-patient transmission during treatment are important issues for substance abuse treatment programs. HBV and HCV have been documented to be transmitted from an infected health care worker or patient through percutaneous or mucosal exposure to blood and other body fluids (CDC, 2003). The opportunity for occupational or nosocomial exposure to hepatitis in a substance abuse treatment program increases as more wraparound services, such as infectious disease testing, immunizations, pain management, and treatment clinical trials, are performed at the treatment site as part of the comprehensive substance abuse treatment plan (Comstock et al. 2004; Krause et al. 2003; Larghi et al. 2002).

This transmission poses personal, legal, and professional risks to patients, health care workers and treatment programs. Providing education—particularly to health care staff on the Occupational and Safety and Health Administration's Blood Borne Pathogens Standard (www.osha.gov/SLTC/bloodbornepathogens/index.html) and use of the latest safety devices—is central to effective efforts to prevention viral transmission. Strict adherence to universal safety precautions is equally important, and dedicated space, equipment, and staff for wraparound services also can significantly reduce hepatitis transmission (Saxena et al. 2003). HAV and HBV vaccination of health care workers and all non-immune individuals in the substance abuse treatment setting also is an important prevention measure and has been shown to be cost-effective (Jacobs et al. 2004).

Protocols for post-exposure treatment and follow-up have been developed (CDC, 2001; Delwaide, 2003; Sounder et al. 2005; West, 2001). For exposure to HBV, the exposed individuals should undergo serological testing. Active (immunization) and passive (anti-HBV antibody) vaccines are effective against HBV infection, if the individual is not vaccine immune, and should be provided within 24 hours of exposure. Antiviral treatment is not recommended for persons diagnosed with acute HBV infection if they have healthy immune systems. Immune globulin and interferon-based antiviral treatment are not recommended for exposure to HCV; instead, the CDC recommends determining the HCV status of the source and the exposed person and follow-up HCV testing to determine if infection has occurred (CDC, 2001).

HCV is detectable as early as 1–2 weeks after exposure, while hepatitis C antibodies can be detected in blood approximately 8 weeks after exposure. Elevations of liver enzymes may be detected 6–12 weeks after exposure (Kim and Saab, 2005). Studies have shown that individuals with symptomatic acute HCV infection have a high likelihood of self clearance of virus or self-cure. The only controlled clinical trial of initiation of interferon-based treatment in acute HCV infection (8 weeks post-exposure) showed a sustained virologic response of 100 percent (Normura et al. 2004). Thus, addressing HCV exposure in acute infection predicts a good outcome either through self-cure or short-term interferon-based treatment.

## Medical Complications and Psychiatric Comorbidities of Hepatitis Infection, Treatment, and Dependence

### Side effects of treatment for hepatitis C infection

Treatment side effects associated with HCV pharmacotherapies have been found to impair quality of life to the extent that approximately 15 percent to 20 percent of patients discontinue treatment. However, as many as 15 percent of patients experience no side effects (Ryder and Beckingham, 2001). Interferon therapy may be associated with side effects such as fatigue, muscle aches, nausea, vomiting, headaches, low-grade fever, and low platelet and neutrophil counts. Although such adverse reactions usually are mild to moderate and can be managed, they may be sufficiently troublesome to influence patient noncompliance or withdrawal from treatment (Table 7; Strader et al. 2004).

Natural and recombinant interferons have short biological half-lives and therefore require daily or thrice-weekly injections. Fluctuations in serum concentrations may undermine both efficacy and tolerance. Pegylated interferon has a longer half-life, a characteristic that improves tolerance and permits less frequent injection. Compared with treatment with interferon alone, interferon plus ribavirin combinations typically result in reduced side effects, with the result that fewer patients discontinue treatment (McHutchison et al. 1998). A recombinant form of interferon, albumin-interferon alfa-2b, has a longer half-life and initial studies show some efficacy in patients who previously failed their interferon therapy (Balan et al. 2006). In these patients, there were no treatment discontinuations due to adverse events and the most common (30–50 percent of patients) side effects of albumin-interferon treatment were headache, fatigue, injection site erythema, and arthralgias.

### Psychiatric illness comorbidity

Manufacturers' package inserts for interferon warn of the potential for neuropsychiatric side effects, the most common being depression and the possibility of psychiatric relapse after beginning therapy. Other neuropsychiatric side effects of treatment include mood alterations, irritability, anxiety, and acute manic episodes (Dell'Osso et al. 2007). Dose-dependent and reversible neuropsy-chiatric effects have been reported to occur in 30 percent to 40 percent of patients during interferon treatment. Treatment with ribavirin may also contribute to interferon-induced depression (Asnis and De la Garza, 2006). The neuropsychiatric comorbidities of interferon alfa-2a or -2b treatment may be severe, limiting treatment in 10 percent to 20 percent of cases and are more than twice as likely in persons with histories of psychiatric disorders than in those without (Davis and Rodrigue, 2001; Ho et al. 2001; Neri et al. 2006). Psychiatric diagnoses, rather than substance-abuse disorders, are a prominent reason for treatment ineligibility unless the patient has been treated and stabilized (Loftis et al. 2006; Muir et al. 1999; Sylvestre et al. 2004). Psychiatric support, including the use of antidepressants, such as selective serotonin reuptake inhibitors or anxiolytics, is frequently required during interferon-based therapy.

Preexisting psychiatric comorbidity has been observed in up to half of all persons entering OTPs (Brooner et al. 1997; SAMHSA, 2005a; Kamal et al. 2007). The management of these illnesses is an important component of the OTPs therapeutic milieu that can promote abstinence and help reduce adverse reactions and risks of drug relapse associated with interferon-based treatments (Ho et al. 2001; SAMHSA, 2005; Kamal et al. 2007). Furthermore, prospective clinical studies demonstrate that a concurrent diagnosis of mental disorder does not preclude effective interferon-based treatment, provided the patient is receiving appropriate psychiatric care and psychotropic drug therapy (Huckans et al. 2006; Pariante et al. 1999; Sylvestre et al. 2004). A study of patients treated with peginterferon alfa-2b and ribavirin while in methadone treatment showed no serious psychiatric events due to interferon-based treatment (Mauss et al. 2004).

An earlier small, prospective, controlled study in Europe examined psychiatric complications during interferon-based combination therapy (Schafer, 2001). Depression increased from baseline in only 16 percent of patients receiving methadone treatment and was found to be mild or moderate. However, during interferon-based treatment, many OTP patients requested increased methadone doses.

The Hepatitis C Resource Centers of the Veterans Administration have produced a monograph—*Management of Psychiatric and Substance Use Disorders in Patients with Hepatitis C: A Reference for Hepatitis C Care Providers*—that provides an algorithm for screening for psychiatric and substance use disorders and addressing suicidal ideation, depression, and post-traumatic stress disorder in the context of substance use and abuse and treatment for HCV infection. The full text can be found at www.hepatitis.va.gov.

### Medication adherence

As with all illnesses treated with prescribed medications, adherence to the HCV treatment regimen is fundamental to a positive treatment outcome. Medication adherence is an important factor in the observation of a sustained virologic response (McHutchison et al. 2001). Multiple recent studies have shown that individuals receiving methadone or buprenorphine treatment, in a supportive medical environment, are adherent to interferon treatment resulting in a high percentage of sustained virologic responses (Krook et al. 2007; Schaefer et al. 2007; Samba et al. 2007; Sylvestre and Clements, 2007). Sustained virologic responses are observed in numerous patients with psychiatric illness with the initiation of psychiatric medications.

In a study of treatment outcomes in a HCV clinical practice, a significantly higher sustained virologic response (53 percent versus 20 percent) was observed in patients who received greater than 80 percent of the recommended dose of interferon-based therapy (Shehab et al. 2004). In an analysis of HCV treatment studies published through 2004 in which patients were treated for HCV infection in combination with substance abuse treatment, sustained virologic response and adherence data in HCV-infected methadone treated patients were comparable to control groups (Schaefer et al. 2004). However, patients with former or current drug abuse were more likely to discontinue treatment early compared to control groups. Patients in methadone treatment have also been shown to discontinue HCV treatment early in the course of treatment (Mauss et al. 2004). Patients who are likely to discontinue treatment early need supportive interventions, including medication adherence interventions (Bacon, 2004). These may include the management of drug-drug interactions and treatment of side effects. Other interventions, such as taking into account lifestyle factors and daily activities or using directly observed treatment protocols, can enhance medication adherence in drug users (Conway et al. 2004; Kresina et al. 2004; Wagner and Ryan, 2004).

Medication adherence interventions are particularly important in the early phase of interferon-based combination therapies. A stronger predictor of a sustained virologic response to combination therapy is the early virologic response—a reduction in detectable HCV RNA during the first 12 weeks of treatment (Fried, 2004). Two studies among patients infected with both HIV and HCV have shown early virologic response to have a value in predicting whether patients will obtain a sustained virologic response through HCV treatment (Ballesteros et al. 2004; Soriano et al. 2004a). Thus, supportive interventions initiated during the first 12 weeks of HCV treatment, such as medication adherence interventions, are particularly important in the medical management of patients with chronic HCV.

Interventions that help patients adhere to treatment are important and necessary in treating of comorbidities associated with substance abuse. Nonadherence with antiviral treatment is common in persons actively abusing substances—particularly alcohol, cocaine, or both—who do not receive adherence interventions (Samet et al. 2004; Weiss, 2004). However, research shows that drug use is not a predictor of noncompliance with treatment regimens and that past drug dependence does not preclude favorable adherence to antiviral therapies (Lucas et al. 2001; Murphy et al. 2004). A prospective longitudinal study of 74 HCV-infected patients receiving interferon treatment, with and without ribavirin, found that adherence was not influenced by sociodemographic factors or source of hepatitis infection. In particular, a history of injection drug abuse was not linked significantly with compliance difficulties (Kraus et al. 2001).

Experience with populations receiving effective substance abuse treatment indicates that stabilized patients tend to be exceptionally compliant, even with unusually burdensome treatment requirements such as reporting to a clinic multiple times each week to receive methadone or to attend therapy groups. Patients in substance abuse treatment programs tend to be resilient, perhaps as a result of the rigors of former addictive lifestyles. With adequate preparation and motivation, they readily endure difficult therapeutic regimens. Furthermore, these patients' frequent contacts with the health care system through OTP's that provide comprehensive services or other treatment venues also promote ongoing compliance monitoring and long-term followup (Backmund et al. 2001; Borg et al. 1999).

### Drug-drug interactions: Addiction pharmacotherapy and HCV treatment

Concerns about therapeutic drug interactions with methadone have been cited as medical justification to exclude patients in substance abuse treatment from receiving treatment for hepatitis and from pharmaceutical trials. Drug interactions, when anticipated and monitored, can be managed by adjustments to medication dosage. Monitoring methadone levels is a critical step in achieving and continuing abstinence in OTPs. Any medication that could alter methadone pharmacokinetics might contribute to drug relapse and modify treatment outcomes.

Methadone and currently used interferon-based treatments do not interact significantly with each other, although the full extent of interactions has not been investigated rigorously. A recent study of 20 adult patients receiving long term methadone treatment showed that peginterferon alfa-2b treatment is associated with minor increases in exposure to methadone that are unlikely to be clinically meaningful (Gupta et al. 2007). A study evaluating interaction between methadone and peginterferon-2a in 24 patients infected only with HCV receiving methadone showed that methadone concentrations were elevated 10 percent to 15 percent but were not clinically significant and that no dose reductions occurred (Sulkowski et al. 2005). There is some evidence that interferon-alfa may mildly inhibit CYP450 enzymes involved in methadone metabolism (Rebetron, 1998). A recent prospective, nonrandomized crossover study of HCV/HIV co-infected patients receiving methadone showed that the pharmacokinetic and the pharmacodynamic properties of methadone were not changed by interferon-alfa-2b treatment (Berk et al. 2007).

Most patients in OTPs who receive methadone also receive prescribed medications for various co-occurring disorders and treatment side effects. Psychotropic agents also often are prescribed to counter the side effects of interferon. Potential drug interactions should be considered carefully to help avoid unintended increases or decreases in methadone concentrations. The number of compounds known to interact with the CYP450 system is large and comedications that influence enzyme activity may lead to decreased methadone concentrations and produce opioid withdrawal symptoms. Conversely, enzyme inhibitors may cause abnormally high methadone levels precipitating toxic adverse reactions (Wolff et al. 2000). For a list of common CYP450 enzyme substrates, inhibitors, and inducers see http://medicine.iupui.edu/flockhart.

Diseases with hepatic involvement could disrupt hepatic metabolic function, down-regulate CYP450 enzymes, and result in slower rates of drug clearance. Viral infections also stimulate cytokine production, which has been associated with suppressed CYP enzyme activity. Thus, conditions suppressing CYP function could produce higher than expected methadone serum levels in HCV-infected patients receiving methadone treatment. However, studies of patient dosing of methadone at OTPs show that patients with HCV infection receive higher methadone doses (Maxwell et al. 2002). However, in one examination of 228 patients receiving methadone treatment, of whom 149 (65 percent) were HCV-infected, no significant differences in dose were found between those who had or did not have HCV (Litwin and Gourevitch, 2001).

Further research is needed to define the interaction of HCV and subsequent liver damage with methadone dose variability. Few studies specifically have measured the effects of hepatitis virus on methadone serum levels. Meanwhile, appropriate monitoring and individualization of methadone dose appear to be essential in patients with HCV infection who are receiving methadone treatment.

### Hepatototoxicity, liver disease, and liver function tests

There are numerous laboratory tests, referred to as liver function tests, which are useful in providing information on liver function/dysfunction. Liver function tests serve as noninvasive markers of liver function and can be used as screening tools to assess liver dysfunction in patients who have unsuspected liver disorders, such as acute viral hepatitis, cirrhosis, or partial bile obstruction. Liver function tests, when performed over time, can also detect a change in liver function or characterize a patterned liver dysfunction. For example, liver function tests can distinguish between liver disorders such as viral hepatitis and cholestatic syndromes. Liver function tests alone cannot be diagnostic for a specific liver disease (Knight, 2005). However, liver function tests allow the health care provider to characterize and follow the course of the liver disease. Although some patients with serious liver disease can have normal test values, liver function tests can be a component of data that may allow the health care provider to predict an outcome early in course of the disease (Peng et al. 2005).

The liver performs hundreds of biochemical/biological functions and, thus, one liver function test cannot accurately assess the total functional capacity of the liver. There are many tests for liver function that can be grouped as follows: tests for the liver's capacity to transport organic anions and metabolize drugs; tests that detect injury or death of hepatocytes (hepatic necrosis); tests of the liver's biosynthetic capacity; and tests that detect chronic inflammation in the liver, altered immunoregulation, or viral hepatitis markers (Kaplan, 1993). A listing of salient liver function tests and their clinical use is presented in Table 8.

Liver function tests are used in multiple ways in the medical management of addiction and the pharmacologic treatment of opioid dependence. Methadone and buprenorphine are metabolized in the liver and tests that measure liver drug metabolism are important in maintaining therapeutic medication levels that do not fall low enough to promote relapse to heroin abuse (Ferrari et al. 2004). Therapeutic drug monitoring for methadone is being investigated for establishing adequate dosing and for monitoring drug diversion (Mercolini et al. 2007). It is routine for determining individual drug pharmacokinetics for patients taking antiretroviral medication for HIV infection (Cone and Preston, 2002; Fraaji et al. 2004).

For injection drug users, determining the presence of viral hepatitis and other underlying liver disease is important in the medical management of opioid dependence. Tests that identify antibodies to hepatitis viruses indicate past exposure of the patient to infection or vaccination. Tests that determine the presence of virus, viral replication, or viral genes and their gene products indicate a current infection with hepatitis virus. Although the liver contains thousand of enzymes, indicators of liver cell injury and acute hepatocellular diseases are the serum levels of aminotransferase enzymes found in hepatocytes. A routine chemistry liver function panel consists of determinations of alanine aminotransferase (ALT) and aspartate aminotransferase (AST), alkaline phosphatase, lactate dehydrogenase, gamma glutamyl transpeptidase, bilirubin (total and conjugated), and albumin levels in a blood sample.

The ALT and AST (and their ratio) are the most frequently measured indicators of liver disease. The measures of serum ALT and AST are an indication of enzyme leakage from tissues rich in the enzymes into the blood. In the case of the liver, the enzymes leak through the damaged cellular (hepatocyte) plasma membrane and, thus, measure cellular damage or death in the liver. Tissues rich in AST are the liver, heart, skeletal muscle, pancreas, and lungs. ALT is primarily present in the liver and kidney; thus, elevated levels of enzymes are not necessarily specific for liver injury or necrosis. However, both AST and ALT are typically elevated in all liver disorders that include acute and chronic hepatitis, cirrhosis, alcoholic liver disease, and liver cancer. Elevations up to eight times the upper limit of normal serum concentrations (Table 8) are nonspecific—they can be found in any of the liver disorders or found in specific patient populations (Celona et al. 2004). The highest elevations occur with liver insults and associated hepatocellular injury, such as drug and alcohol hepatotoxicity or viral hepatitis. Seventy to 90 percent of patients can present with determinations over eight times normal values in liver enzymes. However, as shown in Figure 4, the elevated values may not occur constantly over time in acute or chronic liver disease or even during HIV/HCV coinfection (Maida et al. 2007). Thus, single determinations of liver enzymes may not reflect the level of liver disease. Elevated liver enzymes are considered consistently elevated with a pattern of at least three consecutive monthly elevated readings.

There is a poor correlation between the extent of liver cell necrosis and the elevation of AST and ALT, with the absolute elevation of AST and ALT not a prognostic indicator of liver disease outcome. However, decreases in AST and ALT levels may indicate a liver disease recovery process. In most liver disease, AST and ALT are equally elevated with ALT usually slightly higher than AST. The exception to this observation is alcoholic liver disease in which an AST/ALT ratio of 2 or greater is suggestive of alcoholic liver disease. This is the result of an alcohol-induced deficiency of ALT. Thus, alcohol as a hepatotoxin enhances AST levels but may also reduce ALT levels through cellular metabolic deficiencies (Kaplan, 1993).

As there is a poor correlation between the extent of liver cell necrosis and the elevation of liver enzymes, other laboratory tests have been studied to identify clinical chemistry markers as noninvasive markers of progressive liver disease, especially hepatitis fibrosis (Lichtinghagen and Bahr, 2004; Poynard et al. 2005). These clinical chemistry markers are combined to form multiparameter scores or combined with measures of products of the hepatic extracellular matrix, such as hyaluronic acid; laminin; matrix metalloproteases, or their inhibitors; or collagen degradation products. These studies seek to find biological scores for routine clinical use that are easily obtainable, specific for liver disease, and accurately reflect the stage of liver fibrosis. At a minimum, scores need to accurately differentiate minimal liver disease from advanced liver cirrhosis (Kelleher et al. 2005). With the ability to accurately stage liver disease, biomarkers may replace the current use of liver biopsy in the assessment of liver inflammation and fibrosis due to chronic liver disease (Afdahl, 2004). In addition, serum markers, at that point in the future, may also be used to determine responses to therapy for hepatitis as well as to evaluate disease progression over time (Colletta et al. 2005).

### Liver biopsy and methadone treatment

Liver biopsy remains the “gold standard” or only scientifically proven assessment of liver inflammation and fibrosis resulting from chronic liver disease, including HBV or HCV infection (Gebo et al. 2002a). Thus, liver biopsy is used by medical care providers to determine the grade and stage of liver disease. There are both risks and benefits of a liver biopsy. As with any invasive procedure, the benefits gained from an accurate assessment of liver disease must outweigh the small but definitive risks associated with the biopsy. Risks include bleeding, pain, and puncture of organs, sampling error, patient anxiety, costs, and a low risk of adverse events including death (Sterling, 2005). Contraindications for liver biopsy are an uncooperative patient, impaired coagulation, thrombocytopenia, ascites, biliary obstruction, and vascular tumors. Methadone treatment is not a contraindication for liver biopsy, and studies of HCV and substance abuse have routinely utilized liver biopsy to assess the level of liver disease of patients in methadone treatment (Cournot et al. 2004; Jowett et al. 2001; Van Thiel et al. 2003).

Current HCV treatment recommendations approved by AASLD (AASLD, 2004) are that therapy should be individualized for persons with liver biopsy evidence of no or minimal-to-mild fibrosis, while treatment is indicated for persons with more than portal fibrosis. Because 14 percent to 24 percent of individuals have normal amino-transferases values on liver enzyme panels but show portal fibrosis on liver biopsy, the current HCV treatment recommendations are the following: regardless of the level of aminotransferases, a liver biopsy should be performed when the results will influence whether treatment is recommended, but a biopsy is not mandatory in order to initiate therapy. The value (risk versus benefit) of a liver biopsy has been questioned, (the requirement for a biopsy in Australia was removed in, 2006) particularly for HCV treatment decisions for individuals infected with HCV genotypes 2 or 3, as there is a high likelihood of a sustained virologic response using current standard-of-care treatment regimens (Andriulli et al. 2004; AASLD 2004).

### Continued alcohol use

Alcohol use and alcoholism is a serious medical problem among individuals with opioid dependence (Costenbader et al. 2007; Kosten and O'Connor, 2003; Watson et al. 2007). Heavy alcohol use has been shown in a substantial number of persons first entering OTPs (Chatham et al. 1995; NIAAA 1988; Teplin et al. 2007) as well as individuals in long term methadone treatment (Watson et al. 2007). The Treatment Episode Data Set (TEDS), which summarizes data on admissions to substance abuse treatment programs, reported in 2000 that 23.3 percent of patients entering an OTP indicated the use of alcohol along with heroin (SAMHSA, 2002a). Continued alcohol use can result in decreased medication adherence, drug-drug interactions that modify treatment pharmacokinetics, progressive liver disease, and continued at-risk behavior for infectious disease comorbidity. Excessive alcohol use can reduce the quality of life for those recovering from opioid dependence and lessen their satisfaction with methadone treatment (Senbanjo et al. 2007). Consensus recommendations for medication-assisted treatment are for OTP staff to be trained to recognize the pharmacologic and psychosocial effects of both opioid and nonopioid substances of abuse, including alcohol (SAMHSA, 2005). Thus, patients seeking, entering, or receiving substance abuse treatment for opioid dependence should be carefully screened for alcohol dependence using a validated instrument, such as the CAGE, AUDIT, or MAST questionnaire, with alcohol abuse treatment options available either directly or through referral (Senbanjo et al. 2007; Teplin et al. 2007).

Treatment for alcohol dependence that maximizes good treatment outcomes consists of pharmacotherapy, counseling interventions, and participation in social mutual-help group (Boothby and Doering, 2005; SAMHSA 2005; SAMHSA, 2007). A recent study has shown that individuals receiving methadone treatment substantially reduced heavy alcohol drinking levels when also receiving a brief intervention for alcohol (Watson et al. 2007). Another recent study has shown that methadone can modify the blood alcohol level of individuals who consume alcohol after taking methadone (Clark et al. 2006). In addition, high dose buprenorphine treatment of individuals, who are dependent on both heroin and alcohol, show reductions in use of alcohol as well as heroin (Ciccocioppo et al. 2007). The medical management of withdrawal from alcohol and opiates is complex, and there are substantial differences in severe complications (Kosten and O'Connor, 2003). Medications used in detoxification from one class of addictive drugs may mask symptoms of another class of drugs. Depending on the emergence of serious complications, detoxification of a poly-substance-dependent individual may require inpatient treatment. For individuals who casually use alcohol and who are opioid-dependent, once the patient receives methadone treatment alcohol use has been shown to decrease with time (Caputo et al. 2002).

Patients participating in OTPs have frequently been excluded from fully participating in Alcoholic Anonymous meetings and denied admission to and treatment in traditional addiction and chemical dependency programs (Kipnis et al. 2001). Pilot programs that educate the medical and counseling staff at addiction treatment centers on integrating methadone treatment into the traditional addiction treatment framework have met with success. Thus, patients receiving methadone can complete traditional chemical dependency treatment programs through integration of services at an addiction treatment center (Kipnis et al. 2001).

Alcohol consumption is also a medical issue for patients who are opioid-dependent and have a viral hepatitis infection. Research studies to date have not been able to determine a safe level of alcohol consumption for individuals infected with viral hepatitis. Alcohol consumption of greater than 30 grams/day in men (3–4 drinks, with an average drink comprising 13 grams of alcohol) and 20 grams/day in women increases the risk of liver disease progression and reduces responses to interferon therapy; more than 80 grams/day may seriously compromise HCV treatment (NIH, 2002; Schiff, 1997). For individuals with HBV infection, alcohol consumption has been shown to increase the risk of the development of hepatocellular carcinoma (liver cancer). For those successfully cured of HCV infection, moderate alcohol consumption has been shown to increase the risk of developing liver cancer (Tokita et al. 2005). Alcohol-induced enhancement of viral replication or increased susceptibility of liver cells to viral injury has been suggested as the means through which liver disease may progress (CDC, 1998). The Consensus Conference Statement of the European Association for the Study of the Liver (EASL, 1999) notes that heavy alcohol intake increases HCV viremia and decreases medication adherence with injection drug users at-risk of HCV re-infection.

The most recent NIH and AASLD guidelines acknowledge that HCV treatment for infected injection drug users is feasible and can be effective. However, the guidelines recommend that interferon-based therapy be performed in the context of efforts to address drug/alcohol use, abuse or dependence including participation in OTPs (AASLD, 2004; NIH, 2002). However, continued drug or alcohol use is not a medically valid, or ethically valid (Scott 2005), reason for withholding HCV treatment to individuals in immediate need (AASLD, 2004). As with all medical interventions, HCV treatment decisions need to be made based on an assessment of risks and benefits to the patient. The risk, in this case, is that ongoing alcohol use/abuse enhances liver toxicity resulting in decompensated liver disease or end-stage liver disease. The benefit is amelioration of HCV infection and potential reversal of progressive liver disease.

HCV treatment studies have reported that approximately one fifth of current alcohol/drug abusers do not comply with HCV treatment monitoring or are lost to followup, and ongoing drug use may increase viral load and reduce virologic response to treatment (Davis and Rodrigue, 2001; Sylvestre, 2002). However, patients with co-occurring HCV infection and substance use may complete interferon treatment with careful monitoring and aggressive intervention. Treatment providers must integrate early interventions for drug use and other comorbidities into their HCV treatment algorithm. Using this treatment paradigm, patients with current and past histories of significant substance use disorders are able to successfully complete a course of interferon-based therapy and that sustained virologic response rates are similar to those without such difficulties.

### Liver transplantation

Liver transplantation is standard treatment for individuals with end-stage liver disease. A substance abuse-related diagnosis, either HCV or alcoholic liver disease, is the leading cause (46 percent of cases) and next leading cause (25 percent of cases) for liver transplantation, respectively. The current use of addictive drugs is an absolute contraindication for acceptance into liver transplantation programs (Keeffe, 2000). An abstinence period of at least 6 months is required and patients fully recovered from drug use can be considered for liver transplantation.

A 2001 survey of liver transplantation programs found that 32 percent of programs required patients to discontinue methadone treatment (Koch and Banys, 2001). Approximately 6 percent of all individuals with HCV infection are prescribed methadone. However, 85 percent of all methadone patients are HCV-infected. Studies do not support withholding the provision of liver transplantation from patients receiving methadone, although patients who undergo liver transplantation while receiving methadone do have substantial medical complications. One study described the outcomes of five patients receiving methadone who had been abstinent from illicit drugs and alcohol for at least 6 months. Three patients had end-stage liver disease resulting from HCV infection, one from HBV infection and one from alcoholic liver disease. No patient returned to illicit drug use post-transplantation, and mean patient survival time, at publication, was 1,250 days (over 3 years). The study concluded that acceptable patient survival rates can be achieved in patients receiving methadone as long as patients receive counseling, psychotherapy, and services post-transplantation to enhance retention in care (Kanchana et al. 2002). The largest reported series of patients provided outcomes for 35 patients receiving methadone. The study reported a higher than normal rate of rejection, but patient graft and survival times were comparable to national averages. Eleven percent of patients relapsed to isolated episodes of heroin use post-transplant (Liu et al. 2003).

In a more recent report, 10 patients receiving methadone were compared with 19 nonmethadone, nonopioid-dependent patients, post-transplantation. Patients receiving methadone required significantly more intraoperative anesthesia and postoperative analgesia as well as methadone dose increases (preoperatively compared to postoperatively). Survival time was not different between groups. However, post-transplantation, 20 percent of patients used alcohol or illicit drugs. The authors conclude that liver transplantation patients receiving methadone pose a greater challenge to their medical management but should not be withheld from liver transplantation waiting lists (Weinrieb et al. 2004). Other case reports of patients receiving methadone treatment and a liver transplant show 5 years post-transplant that the patients are stable and continuing on methadone treatment (Hancock et al. 2007). Information on the organ transplantation process and waiting lists for transplantation can be found at the United Network for Organ Sharing Web site www.unos.org.

### Hepatitis-HIV co-infected patients and liver transplantation

Co-infection of hepatitis and HIV viruses greatly accelerates the progression of liver disease associated with viral hepatitis. As an emerging problem, persons with co-infection face insurmountable obstacles to treatment or transplantation for their liver disease (Nadler, 2001; Stock and Roland, 2007). Life expectancy in HIV-infected individuals has been extended due to advances in antiret-roviral therapy, and HIV infection now can be considered a chronic illness, rather than an absolute exclusion to organ transplantation. Liver transplantation is being evaluated as a therapeutic option for patients with controlled HIV infection and end-stage liver disease resulting from HCV infection, HBV infection, or drug-induced hepatotoxicity (acute liver failure). Liver transplants have been successfully performed in at 11 centers worldwide between 1990 and 2001. The one caveat has been the potential for drug interactions between anti-retroviral agents and immunosuppressive drugs and the need for dose adjustments.

Clinical trials at 10 centers in the United States are underway to assess the impact of HIV infection (and co-infection with HCV or HBV in some patients) on liver transplant outcomes. While relapse of HCV infection is common after transplantation, initial reports indicate that transplantation is effective with an 85-percent, 1-year survival rate, which is similar to the non-HIV HCV-infected patient survival rate (Castells et al. 2006; Neff et al. 2005).

The accumulated experience in Europe and the United States indicates that the 3-year survival in HIV-positive liver transplantation recipients is similar to that of HIV-negative recipients. Guidelines for the selection of patients with HIV-infection for liver transplantation have been generated in the United Kingdom and Spain (O'Grady et al. 2005; Miro et al. 2005). Methadone treatment is not an exclusion criterion.

### Diabetes, kidney disease, opioids, and kidney transplantation

**Figure 10:**
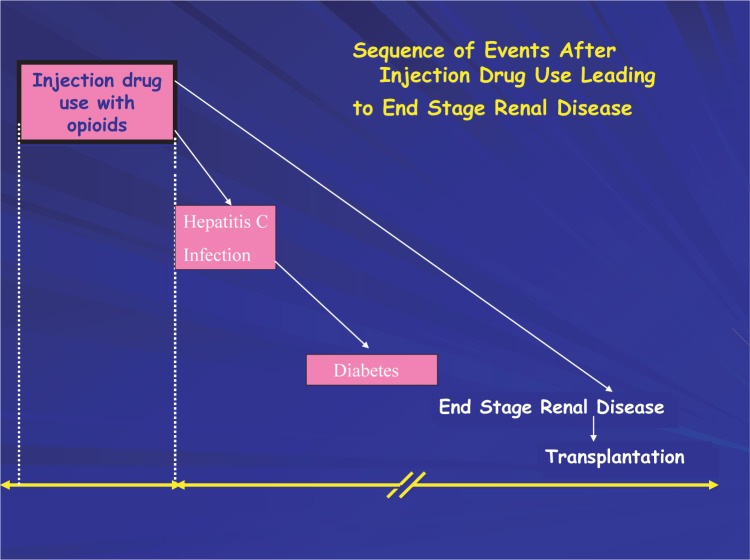
Enhancement of heroin-associated nephropathy by HCV infection and diabetes.

Injection drug use, HCV infection, and type 2 diabetes mellitus each independently, and in concert, can result in chronic kidney (renal) disease and the need for transplantation. Data gathered through NHANES III show that the prevalence of type 2 diabetes in adults aged 20-59 with HCV infection was 3.4 percent (Behrendt and Ruiz, 2005). HCV infection and type 2 diabetes was associated with a family history of diabetes, as well as advanced liver fibrosis, but not the classical phenotype for diabetes (overweight individuals with coronary heart disease). HCV infection has been associated with insulin resistance among older adults and those at risk of HIV infection (Howard et al. 2007). Opium addiction has been shown to enhance the metabolic abnormalities associated in patients with type 2 diabetes (Karam et al. 2004). Numerous studies have described the clinical and pathological features of renal disease associated with injection drug use of heroin, cocaine, morphine, amphetamine, and other narcotics (reviewed in Dettmeyer et al. 2005). Drug addiction neuropathy, including renal failure associated with oxycodone addiction, constitutes an important cause of end-stage renal disease that can be augmented by a genetic predisposition to diabetes as well as HCV infection (Hill et al. 2002). Thus, for the injection drug user seeking treatment, there is a spectrum of diseases and infections that can exacerbate addiction-associated renal disease leading to end-stage renal disease (Fig. 10).

Methadone has been shown to be safe and effective in patients with renal disease who are undergoing dialysis (Dean, 2004). Renal transplantation of HIV-positive patients has been performed (Kumar et al. 2005; Roland, 2004). Post-transplant survival has improved in these individuals with the use of antiretroviral therapy to control HIV infection. Current experience in renal transplantation in HIV-infected patients in the United States indicates that the 3-year survival rate is similar to that of HIV-negative transplant recipients, with virological and immunological control of the infection by anti-retroviral treatment. There has been no increase in the number of opportunistic infections or tumors due to immunosuppression post-transplantation. The criteria for selecting HIV-positive transplantation candidates include no opportunistic infections, CD4 cell counts greater than 200 per μl. and control of HIV infection with an undetectable viral load. In Spain, where most of these patients are former drug abusers, a 2-year period of abstinence from cocaine and heroine abuse is also required, although patients are permitted to participate in methadone treatment (Trullas et al. 2005). Problems post-transplantation include interactions between anti-retroviral drugs and immunosuppressive drugs, management of HCV coinfection, and progressive liver disease, as well as acute graft rejection.

### Dependence and immune modulation

Both basic research studies and clinical observational studies indicate that opiates and opiate abuse have a broad influence on immune networks and their function (Donahoe and Vlahov, 1998; Sharp, 2003). Opioid abuse may alter immune function (Alonzo and Bayer, 2002; Ryan et al. 2004). Abnormalities have been observed in immune responses of heroin injection drug users, including diseased lymph glands, elevated white cell counts, increased antibodies, and false-positive tests for syphilis, rheumatoid arthritis, and other illness. T cells, mediators of cell-mediated immunity, have been shown to express cell surface opioid receptors (Sharp, 2003). These receptors, when bound by opioids, would reduce the immune response induced by T cells, thereby suppressing overall immune responses.

Clinical investigations demonstrate that immune response abnormalities can be moderated or eliminated by methadone treatment (Donahoe, 1993; Novick et al. 1991; SAMHSA, 1993; Sergio et al. 2007). Methadone treatment has been reported to normalize immune function and stress responses in former injection drug users (Zajicova et al. 2004). Research also suggests however, that inadequate methadone-maintenance doses or withdrawal from methadone may create extraordinary stress, potentially altering immune system function (McLachlan et al. 1993).

### Dependence and cancer

One out of every two men and one of three women in the United States develop cancer at some point in their lifetime (DHHS, 2005). Cancer arises from a loss of the normal regulatory control of how and when cells grow, divide, and proliferate. The loss of the regulatory control of cellular and division is a multistep process called carcinogenesis and has a strong genetic component (Hall et al. 2001). However, research studies show that environmental factors, such as lifestyle behaviors, can contribute to the process of carcinogenesis and, for instance, trigger the malignant transformation of a precancerous lesion to form cancer. A well-recognized behavior that is closely associated with an increased risk of cancers in various organs of the body is tobacco use resulting from nicotine addiction (Nishikawa et al. 2004). Chronic alcoholic beverage consumption is also a significant risk factor for cancer of the digestive tract (oral-pharynx, larynx, esophagus, liver, and colon) as well as breast (Poschl et al. 2004). Alcohol consumption in association with cigarette smoking, which may occur in a significant number of individuals receiving substance abuse treatment, may have a significant impact on carcinogenesis (Poschl and Sietz, 2004). The additive or synergistic effect of two or more agents leading to cancer is termed co-carcinogenesis. Co-carcinogenesis has been proposed for virus-chemical interactions to cause cancer. Nicotine and/or alcohol may be important co-carcinogens in patients with substance use disorders, particularly if these patients are infected with HBV or HCV.

The Eleventh Edition of the Report on Carcinogens (DHHS, 2005; available at http://ntp.niehs.nih.gov) has for the first time listed HCV and HBV as known human carcinogens. Both HBV and HCV infections are listed as a cause of liver cancer. Thus, substance abuse treatment programs that address lifestyle behaviors such as smoking and alcohol consumption, as well as infections related to IDU such as HBV and HCV, are preventing the occurrence of additional comorbidities in their patient population. Primary prevention programs for cancer are focusing on lifestyle changes, which include diet and exercise, to promote better health for their patients (Festi et al. 2004; Martinez, 2005).

## Resources for Care and Treatment

### Medical education, access to care and treatment, and multidisciplinary service teams

Hepatologists, gastroenterologists, infectious disease specialists, primary care providers, general and family practitioners, psychiatrists, and addiction treatment specialists would benefit from continuing medical education related to the care and treatment of chronic HCV infection in injection drug users. Further understanding of substance dependency and the stages of addiction recovery, particularly with respect to methadone and buprenorphine treatment, are needed. Substance abuse treatment programs need to address hepatitis infection, liver disease, and provide community resources to support patient care and treatment. Information and education resources are readily available on the Internet. A listing of salient resources is provided in Table 9.

The development of multidisciplinary service provider teams and regular interaction among addiction-treatment providers, liver-treatment specialists, infectious disease specialists, primary care providers, case managers and patient advocates as well as peer outreach workers has been recommended to better coordinate hepatitis testing, patient care, and access to care. For injection drug users receiving care in an urban primary care clinic, only 55 percent were tested for hepatitis infection (Trooskin et al. 2007). Follow-up medical care of individuals testing positive for exposure to hepatitis infection is essential. For HCV-positive injection drug users receiving a referral for medical care, a recent study has shown that only 24 percent returned for that care (Reynolds et al. 2006). Of that small group, less than half further sought specialty care for liver disease. Factors that predicted further seeking care included participating in residential drug treatment. Referral to a hepatology clinic for HCV patients receiving methadone treatment was more successful, with 63 percent of patients attending followup appointments (Hallinan et al. 2007). Public health-sponsored hepatitis testing programs report similar referral success, with 70 percent of those tested contacted after HCV testing, 55 percent of those contacted receiving a medical evaluation, and 12 percent receiving HCV care and treatment (Mark et al. 2007).

To foster improved testing, access to care, and treatment for hepatitis, a cooperative and interactive approach is necessary. Components of this approach include interventions to promote testing and reduce barriers to care and interventions to promote adherence to medication regimens, treatment for coexisting physical and psychiatric conditions, and implementation of strategies to prevent relapse during and following treatment (Davis and Rodrigue, 2001; NIH, 2002). Integrating delivery of substance abuse treatment with prevention, care and treatment of liver disease has been demonstrated as an important strategy for improving attendance at medical visits and optimizing treatment outcomes (Litwin et al. 2005; Muir et al. 1999; Sylvestre and Zweben, 2007; Willenbring et al. 2004).

### Models of co-location of prevention, care, and treatment services

The co-location of medical/health services or so-called “one stop shopping” for health care results in an increased utilization of services, enhanced medical outcomes and patient statisfaction, as well as enhanced knowledge, communication, and collaborations among co-locating groups (Jackson et al. 2007). For services related to substance abuse treatment and the prevention, care and treatment of hepatitis infection, co-location of services can occur either in the substance abuse treatment setting or in the primary care/medical setting. Regardless of the site, integration of services has been shown to enhance the use of services for those who are medically disadvantaged as well as provide a venue for addressing gender issues in the context of substance abuse treatment (Ayalon et al. 2007; Najavits et al. 2007; Passey et al. 2007).

#### Co-location of services in a substance abuse treatment setting

Co-location of HCV and HIV prevention, care, and treatment services, as well as other wrap around services, in substance abuse treatment settings fosters access to care for patients with multiple comorbidities, many of whom likely would not be accessing needed care (Dore and Thomas, 2005; Gunn et al. 2005; Kresina et al. 2005). Co-location of services has also been shown to lead to high rates of adherence with liver biopsy and initiation of antiviral therapy (Litwin et al. 2005). Many substance abuse treatment programs are developing model programs of integrated service delivery to significantly improve patient outcomes for this difficult-to-treat patient population. To facilitate this process the American Liver Foundation has developed *HIT'M*, a manual for training staff to integrate hepatitis prevention into HIV/AIDS, STD, and drug treatment programs.

In the United States, methadone treatment is federally regulated though the Code of Federal Regulations which require OTP's to dispense methadone dosages in a highly rigorous and structured fashion (CFR, 2001). However, these regulations do not preclude the integration of medical and social services to provide a comprehensive therapeutic milieu, comprising primary medical care, psychosocial and infectious disease counseling, vocational rehabilitation, ongoing performance monitoring, street outreach, prevention services and patient education. Prevention, care, and treatment programs are being implemented that integrate general and HCV and/or HIV-related medical, prevention, or mental health services into methadone treatment programs (Gourevitch et al. 2007; Litwin et al. 2005; McLaughlin, 2007; Strauss et al. 2005; Sylvestre et al. 2004). Pilot model programs range from interventions, such as smoking cessation or case management (Sorensen et al. 2006; Stein et al. 2006) to the development of methadone health care provider teams that include a physician (internist or family practitioner), a mid-level provider (PA or NP), psychiatrist, a social worker/case manager, nurse, substance abuse treatment counselors with peer support groups. The co-location of medical care with methadone treatment has been shown to reduce emergency department and inpatient care, increase ambulatory patient care with no net increase in expenditures (Gourevitch et al. 2007). The co-location of buprenorphine treatment with methadone treatment and a chemical dependency program that in combination provide substance abuse counseling, social services, medical and psychiatric treatment has shown to maintain patient retention and abstinence (Whitley et al. 2007).

#### Co-location of services in a primary care or HIV care and treatment setting

In the United States, the regulation of methadone treatment prohibits the implementation of methadone treatment in a primary care setting. However, a model program received a regulatory exemption and provided methadone treatment through a public hospital for a period of one year (Merrill et al. 2005). An evaluation of the program revealed high patient statisfaction and retention in the program, enhanced medical service utilization by the patients and a change in medical provider stigma toward methadone. In countries where methadone is available in a primary care setting, two recent studies (Grebely et al. 2007a; van Beek, 2007) have show that successful treatment of HCV or HIV infection using directly observed therapy in a primary care setting that treats injection drug users with methadone.

Pharmacotherapy for injection drug users in a primary care setting is available in the United States in the form of treatment with buprenorphine. ‘Real world' clinical practice of using buprenorphine to treat opioid dependent patients in a primary care practice are reported as good, with only 32 percent of patients dropping out of treatment from 2003–2005 (Magura et al. 2007). Mainly through the funding of the Health Services Resources Administration (HRSA), numerous pilot projects have been initiated to develop the best clinical practices that integrate buprenorphine treatment regimens into HIV primary care programs (Altice et al. 2006; Kresina et al. 2005a; Lum and Tulsky, 2006; Mitty et al., 2007; Sullivan et al. 2006; Taylor, 2005; Turner et al. 2005). Integrating care for HIV infection and treatment for opioid dependence optimizes outcomes for patients with both disorders. In addition, buprenorphine treatment in primary care is associated with a reduction in drug-related HIV risk (Sullivan et al. 2007). Other model programs have shown that buprenorphine treatment is effective in homeless opioid dependent patients despite their social instability, greater comorbidities, and severe chronic drug use (Alford et al. 2006). However, barriers to adopting buprenorphine treatment in HIV primary are many and include policy and finance issues as well as infectious disease physician access to substance abuse treatment experts (Cunninghan et al. 2007; Schackman et al. 2006; Turner et al. 2005).

#### Outreach, referral networks, and community programs

Individuals who abuse heroin, cocaine and alcohol frequently utilize emergency departments for needed health care (O'toole et al. 2007). Developing programs that address the underlying needs of individuals who use emergency departments for medical care and linkage to substance abuse treatment can reduce hospital use (Friedman et al. 2006). Drug treatment linkage strategies vary but both vouchers and case management have been shown to be effective in enhancing access to drug treatment services, including methadone treatment programs (Alexander et al. 2007; Barnett et al. 2006; Sorensen et al. 2005; Zaller et al. 2006). A referral intervention for women that promoted quality of services also enhanced access to outpatient drug treatment (Passey et al. 2007). Referral programs from sexually transmitted disease clinics that offer an initial screen for drug use has also been shown to be successful (Yu et al. 2007). Linking methadone treatment programs to prison release programs can address the high risk of overdose and disease transmission in recently released inmates for (McMurran, 2007; Rich et al. 2005).

Community programs can also enhance the use of medical services for the prevention, care and treatment of substance abuse and hepatitis infection. Programs can utilize peer outreach workers/educators to enhance knowledge of hepatitis infection and access to prevention and care (Sylvestre ands Zweben, 2007). Community interventions that address risk reduction motivation and behavioral skills in the context of pharmacotherapy for opioid dependence have been shown to be successful (Copenhaver and Lee, 2006). Community-based partnerships comprising community teams can recruit participants for family-based drug treatment and prevention interventions (Spoth et al. 2007).

Other community based programs can involve state and county health departments for the provision of resources to support HCV services. For example, in Oregon, the Multnomah County Health Department Viral Hepatitis C Integration Program is dedicated to building capacity within the county health department and in the community through integrating services and creating community services and resource linkages. Five primary areas are targeted HIV prevention and outreach to build street outreach and education for hepatitis; sexually transmitted disease clinics to implement HCV testing, post-test counseling, and hepatitis A and B vaccinations; social work for individuals who test positive for HCV, short-term case management, and social service support; development of a referral system among primary care and family services addiction specialists; health education for the development and dissemination to the community of hepatitis curricula; and community planning processes to establish community planning groups for development and implementation of a HCV strategic plan. In this program, consumers of hepatitis services are part of the community planning process and interface with institutional decision makers and the county health department policymakers. Academic health centers can provide novel programs to address the medical management of chronic hepatitis infection through a partnership of nurse practitioners, primary care physicians, physician assistants, pharmacists, psychiatrists, and substance abuse treatment providers (Arora et al. 2007).

Community planning and health services referral networks are fundamental to providing needed medical and social services not available at OTPs (Fletcher et al. 2003). To help achieve a viable referral network, substance abuse treatment programs need to identify health care providers in the community willing and trained to provide medical and social services to their patients (Novick, 2000; Sweeney et al. 2004). Greater improvement in post-treatment outcomes has been shown in programs that tailor frequency and type of service to unique client needs (Rowan-Szal et al. 2000). Substance abuse treatment programs should be advocates for their patients needing and seeking care and treatment, thereby providing a support network throughout the course of therapy.

## Summary

Individuals receiving pharmacologic therapies for opioid abuse/dependence often have co-occurring disorders that can complicate substance abuse treatment regimens. Addressing co-occurring disorders and co-infections in a comprehensive fashion through screening, testing, and medical management can facilitate successful patient outcomes of substance abuse treatment.

Outreach workers and peer advocates working with health care providers should encourage HCV-infected injection drug users to access treatment for opioid abuse/dependence whether or not they are receiving treatment for HCV infection. Treatment for chronic HCV infection has been successful when patients are provided daily methadone and methadone treatment has been shown to reduce risky behaviors that can spread HCV infection. Limited data are available on the treatment of viral hepatitis infections for patients who inject drugs and who are not in drug treatment programs with reported clinical outcomes less successful.

The decision of whether to treat a patient for hepatitis infection should be made considering the anticipated risks and benefits for the individual. Treatment for hepatitis infections should not be denied to any patient needing it, and efforts encompassing proven supportive interventions may be required for these individuals to become ready for treatment. Individuals stabilized through treatment using methadone or buprenorphine and otherwise well engaged in an opioid treatment program are candidates for treatment for chronic HCV infection. Physicians are not required to cease treatment for opioid dependence in order to begin treatment for HCV infection. Substance abuse treatment programs that provide comprehensive services for drug users in a supportive/accepting environment promote compliance with therapeutic treatment regimens. These programs can facilitate medical follow-up and foster social, psychological, and vocational rehabilitation as well as a viable way to prepare injection drug users for pharmacotherapies for chronic liver disease and help promote medication compliance and favorable treatment outcomes. Patients receiving medication for opioid dependence through office-based treatment services may require treatment for hepatitis infections during their substance abuse treatment. Therefore supportive environments that promote hepatitis prevention, care, and treatment programs need to be available and integrated into all venues providing pharmacotherapy for opioid dependence.

## Figures and Tables

**Table 1:** Disease burden from viral hepatitis in the US in 2006.

Virus	Infections		Comment
	New	Chronic	
A (HAV)	32,000*	none	No chronic disease; one-third of persons in the United States have evidence of past HAV infection
B (HBV)	46,000* acute	1.25 million acute	About 5 percent of persons in the United States have evidence of past or current HBV infection
C (HCV)	19,000*	3.2 million	About 1.6 percent of Americans have evidence of past or current HCV infection
D (HDV)	Unreliable		Only concurrent with HBV infection

* Estimated new infections in 2003. Data are influenced by annual incidence fluctuations and variable estimates of unreported and/or undiagnosed cases.

Data are from www.cdc.gov/ncidod/diseases/hepatitis/resource/dz_burden.htm

**Table 2:** Factors influencing HCV treatment outcomes.

Host factors	Viral factors
• High degree of fibrosis	• Genotype 1
• Age >40 or 50 at time of infection	• Viral load > 2 million copies/ml
• Male sex	
• Weight >75 kg; 165 lbs	• Large number of quasispecies (a measure of the HCV genome heterogeneity)
• African-American	
• Long duration of infection	
• HIV co-infection	

**Table 3:** Treatment targets and possible options for patients with HBV/HIV co-infection.

Co-infection target	Treatment option
Hepatitis B virus (HBeAg^+^)	interferon-a (pegylated); entecavir; or Adefovir
Hepatitis B virus (HBeAg^−^)	Interferon-a (pegylated); Entecavir; Adefovir
Hepatitis B virus and HIV	antiretroviral (HIV) regimen, including lamivudine or emtricitabine with tenofovir or adefovir
To control HIV	antiretroviral (HIV) regimen + adefovir or entecavir antiretroviral (HIV) regimen + one HBV antiviral drug to avoid immune reconstitution induced liver disease

**Table 4:** Comorbidities associated with substance abuse and dependence.

• ***Medical***: HCV infection, HBV infection, tuberculosis and other pulmonary disease, immune deficiency, human immunodeficiency virus infection, sexually transmitted diseases, sexual disorders, dental and periodontal disease, nutrient deficiency, cardiovascular disease, sleep disorders, chronic pain syndromes
• ***Psychiatric***: Axis I spectrum disorders such as depression, anxiety, post-traumatic stress disorder, personality disorder, bipolar disorder, attention deficit hyperactivity disorder, schizophrenia, cognitive dysfunction; Axis II personality and developmental disorders
• ***Social***: poverty, homelessness, family dysfunction, corrections/prison, violence, sexual abuse, drug-using peer groups, easy drug access, lack of occupation and skills
• ***Other Addictions and Abuse***: alcohol, nicotine, stimulants, cocaine, hallucinogens, marijuana, prescription drug, internet, gambling

**Table 5:** Pharmacotherapy and behavioral therapy comprising a comprehensive substance abuse treatment plan.

***Pharmacotherapy***
• Opioid Dependence Methadone—Federally regulated through OTP; opioid receptor agonist for pharmacological therapy Buprenorphine—office-based opioid treatment or OTPs; Federally regulated, partial opioid receptor agonist for pharmacological therapy Naltrexone—office-based and substance abuse treatment programs; used when opioid abstinence is possible without significant relapse risk; opioid receptor antagonist for relapse prevention
• Alcohol Dependence Naltrexone—an “anti-craving” agent, opioid receptor antagonist; reduced reward effect with daily use; new forms are long acting Acamprosate—an “anti-craving” agent that normalizes glutamatergic neurotransmission; slow acting, attenuates relapse Disulfiram—a “vicarious” aversive medication supporting complete abstinence to alcohol that blocks complete oxidation of alcohol with accumulation of acetaldehyde and resultant unpleasant “allergic” physical symptoms when alcohol is absorbed (e.g. flushing, headache, and vomiting)
• Nicotine Dependence Nicotine replacement therapy—many over-the-counter regimens, such as patches, gum, and inhalers, are used to replace the daily physical requirement for nicotine and may be used for nicotine withdrawal or maintenance Buproprion—an antidepressant also found to be an “anti-craving” agent that reduces the psychological craving for tobacco
***Behavioral therapy***
• Brief interventions for 1 to 3 visits (low intensity); for early drug use and substance abuse; available in many different outpatient settings
• Motivational enhancement interviewing and therapy
• 12-step facilitation
• Stage-of-change model interventions
• Long-term, multimodal, and multidimensional comprehensive therapies and interventions to restructure belief and cognitive systems; enhance coping strategies; and change friendships, environment, and behavior
• Individual interpersonal one-on-one therapy, such as cognitive behavioral therapy and insight-oriented psychotherapy
• Group therapy—such as family or faith-based, Therapeutic Communities
• “12-step” programs and “clean and sober” recovery living environments in which peer groups interested in sobriety mutually help one another stay sober

**Table 6:** Salient Hepatitis C curricula developed by federal and state agencies.

Agency	Program and website
Substance Abuse and Mental Health Services Administration (SAMHSA)	HIV and Hepatitis Matrix www.samhsa.gov/Matrix/matrix_HIV.aspx American Association for the Treatment of Opioid Dependence web site www.AATOD.org/hepatitis.html
Centers for Disease Control and Prevention (CDC)	The Division of Viral Hepatitis—Hepatitis C Toolkit, brochures, posters, Counseling and Training resources www.cdc.gov/ncidod/diseases/hepatitis/c/#materials The National Commission on Correctional Health Care Curriculum www.cdc.gov/ncidod/diseases/hepatitis/resources/training/ncchc_man.htm
Department of Veterans Affairs (VA)	VA National Hepatitis C Program Educational resources. Patients corner, VA information www.hepatitis.va.gov
Health Resources and Services Administration (HRSA)	Integrating HIV and HCV services www.hab.hrsa.gov/catie/list.asp?ref=256
New York City Department of Health and Mental Hygiene	Understanding Hepatitis C www.nyc.gov/health
Massachusetts Department of Health	Massachusetts Hepatitis C Program www.mass.gov/dph/cdc/masshepc/default.htm
New York State Department of Health	National Virus Hepatitis Training
Texas Department of State Health Services	Texas Hepatitis C Initiative Counselor Training Manual www.tdh.state.tx.us/ideas/hepatitis Also required continuing nursing education through the CDC, Texas Nurses Association or National Center of Continuing Education www.tdh.state.tx.us/ideas/hepatitis/heaptitis_c/professional/cne

**Table 7:** Common side effects of interferon-alpha and ribavirin.

**Flu-like Symptoms**- fever, headache, myalgia, fatigue, asthenia, rigors, dizziness, influenza-like symptoms
**Gastrointestinal**- diarrhea, anorexia, nausea, vomiting, abdominal pain
**Neuropsychiatric**- depression, impaired concentration, irritability, insomnia
**Skin/Appendages**- alopecia, pruritus, rash
**Hematologic**- decreased hemoglobin, decreased white blood cell count, decreased platelet count

**Table 8:** Common liver function tests and their clinical value.

Liver function test	Normal value*	Clinical value
Alanine aminotransferase (ALT)	10–31 U/L	ALT lower than AST in alcoholism
Albumin	3.5–5.2 g/dL	Assess severity/chronicity measures liver protein synthesis
Alkaline phosphatase	25–112 U/L	Diagnose cholestasis and hepatic infiltrations
Aspartate aminotransferase (AST)	10–44 U/L	Early diagnosis and monitoring of hepatic necrosis
Bilirubin (Total)	0.1–1.0 mg/dL	Assess severity of cholestatic liver disease
Gamma glutamyl transpeptidase	16–74 U/L	Diagnose alcohol abuse Marker of cholestasis
Prothrombin time	11.3–16.5 sec	Assess severity of liver disease; measure of liver protein synthesis

* Normal values vary with the laboratory reporting them (adapted form Larson et al. 2005).

**Table 9:** Salient internet resources: methadone, Hepatitis, co-infection.

•Addiction Treatment Forum www.ATForum.com •American Association for the Study of Liver Diseases (AASLD) www.aasld.org •American Association for the Treatment of Opioid Dependence (AATOD) www.AATOD.org/ •American College of Gastroenterology (ACG) www.acg.gi.org • American Gastroenterological Association www.gastro.org/ • American Liver Foundation (ALF) www.liverfoundation.org • American Society of Addiction Medicine (ASAM) www.asam.org • Centers for Dis. Control and Prevention (CDC) www.cdc.gov/ncidod/diseases/hepatitis • Center for Substance Abuse Treatment (CSAT), SAMHSA ww.samhsa.gov/centers/csat2002/csat_frame.html • Cleveland Clinic Foundation. Hepatitis C Management www.clevelandclinicmeded.com/hcv • Department of Veterans Affairs. Hepatitis C Resource Centers. www.hepatitis.va.gov • Division of Pharmacologic Therapies, CSAT, SAMHSA www.samhsa.gov/centers/csat/content/dpt/index.html • GastroHep.com www.gastrohep.com • Hepatitis B Foundation www.hepb.org • Hepatitis c Advocacy www.hepcadovcacy.org • Hepatitis C Connection www.hepc-connection.org • Hepatitis C in Me (Maine) www.hepatitiscnme.org • Hepatitis Foundation International (HFI) www.hepfi.org • HIV and Hepatitis. www.hivandhepatitis.com	•Infectious Disease Society of America. www.idsociety.org/content/navigationMenu/Practice_Guidelines/Guidelines_by_topic/hepatitiscLibrary, Alcohol and Drug Abuse Institute, University of Washington http://lib.ada.washington.edu • Matrix Institute on Addictions www.matrixinstitute.org • Medscape HIV/AIDS from WebMD www.medscape.com • National Alliance of Methadone Advocates www.methadone.org/ • National Center for Complementary and Alternative Medicine (NCCAM), NIH nccam.nih.gov • National Digestive Diseases Information Clearing-house (NDDIC), NIDDK, NIH www.niddk.nih.gov • Natl. Foundation for Infectious Diseases (NFID) www.nfid.org/ • National Institute of Diabetes and Digestive and Kidney Disease (NIDDK), NIH:www.niddk.nih.gov/health/digest/pubs/hep/ • National Institute on Alcohol Abuse and Alcoholism, NIH www.niaaa.nih.gov/ • National Institute on Drug Addiction, NIH www.nida.nih.gov/ • National Institutes of Health (NIH) www.nih.gov/ • New York Department of Health AIDS Institute Substance Use Guidelines http://hivguidelines.org/public_html/center/clincial-guidelines/sub-gl/substance.html • Organization to Achieve Solutions in Substance Abuse (O.A.S.I.S.) www.oasisclinic.org/ • Projects in Knowledge. Initiatives in Gastroenterology www.projectsinknowledge.com/recent/indexG.html • Texas Department of State Health Services-Public Information kit-Hepatitis C Initiative www.tdh.state.tx.us/ideas/hepatitis/hepatitis_c/overview/public_info_kit/ • University of California, San Francisco Center for Information. Coinfection with hepatitis and HIV http://hivinsite.ucsf.edu/

All sites were accessed and active as of September 2007.
